# Floating Interlayer and Surface Electrons in 2D Materials: Graphite, Electrides, and Electrenes

**DOI:** 10.1002/smsc.202100020

**Published:** 2021-07-17

**Authors:** Takeshi Inoshita, Susumu Saito, Hideo Hosono

**Affiliations:** ^1^ Materials Research Center for Element Strategy Tokyo Institute of Technology 4259 Nagatsuta Kanagawa 226-8503 Japan; ^2^ International Center for Materials Nanoarchitectonics National Institute for Materials Science Tsukuba Ibaraki 305‐0044 Japan; ^3^ Research Center for Functional Materials National Institute for Materials Science Tsukuba Ibaraki 305‐0044 Japan; ^4^ Department of Physics Tokyo Institute of Technology 2-12-1 Oh-okayama, Meguro-ku Tokyo 152-8551 Japan

**Keywords:** 2D materials, electrides, graphite, layered materials, surface state, work functions

## Abstract

Over the last half century, layered materials have been at the forefront of materials science, spearheading the discovery of new phenomena and functionalities. Certain layered materials are known to possess electronic states unassociated with any of the constituent atoms, having a large proportion of their probability amplitude in the space between the layers. Usually, such a nucleus‐free interlayer state has energy above the Fermi level and is unoccupied. However, the energy decreases when cations are intercalated and may cross the Fermi level, as in the case of C_6_Ca, a superconductor with a *T*_c_ of 11.5 K. A major thrust to the research of interlayer electrons comes with the discovery of layered electrides, which are alternating stacks of positively charged ionic layers and negatively charged sheets of electrons in the interlayer space. When intercalation compounds and layered electrides are thinned down to the atomic scale, the interlayer states survive as surface states floating over the surface. This review provides a unified overview of the two classes of materials hosting interlayer floating electrons near the Fermi level, intercalation compounds and layered electrides, and their properties, including high electron mobility, low work function, ultralow interlayer friction, superconductivity, and plasmonic properties.

## Graphical abstract

Many layered materials host interlayer states with electrons residing mainly in the interlayer space. Such states usually have energy higher than the Fermi level and are empty. However, in intercalation compounds and layered electrides, these states come down in energy, with the help of attraction by nearby cations, and can be populated by electrons to show many enticing properties.

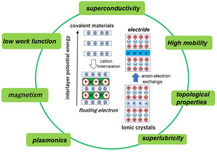

## 1 Historical Background

When discussing the electronic properties of matter, researchers have conventionally adopted the linear combination of atomic orbitals (LCAO) picture, which starts from the valence orbitals of the constituent atoms and lets the orbitals overlap to form energy bands of finite width. Even for simple *sp* metals, for which the nearly free electron (NFE) model works well, their energy bands are often interpreted in association with underlying atomic orbitals. Therefore, it came as a big surprise when Posternak et al. reported in 1983, based on their electronic structure calculation, that graphite intercalation compounds (GICs) host a free‐electron‐like band having its density mainly in the 2D interlayer space in addition to the bands derived from atomic orbitals.^[^ ^1^ ^]^

These bands had already been reported in preceding papers but were interpreted erroneously as arising from the metal *s* orbital because of their parabolic dispersion.^[^ ^2^, ^3^ ^]^ Posternak and co‐workers subsequently showed that such bands, which they named interlayer bands (ILBs), exist even in unintercalated graphite or hexagonal boron nitride (h‐BN),^[^ ^4^ ^]^ albeit at a higher energy, and drop toward (and eventually intersect) the Fermi energy (*E*_F_) when intercalated with cations. ILBs were met initially with suspicion but accepted gradually, as they were confirmed both theoretically and experimentally.^[^ ^5^, ^6^, ^7^, ^8^ ^]^

Similar nonnuclear floating electrons were found to also exist at the surfaces of these materials, about 2 Å from the surface atomic layer.^[^ ^9^ ^]^ While there are materials such as C_8_K having an ILB intersecting *E*_F_, ILBs usually appear a few eV above *E*_F_ and do not contribute to properties such as electronic conduction. Perhaps, for this reason, no further attention was paid to ILBs over the ensuing years.

It was in 2005 that ILBs came under a fresh spotlight with the discovery that C_6_Ca and C_6_Yb superconduct with the highest transition temperatures (11.5 and 6.5 K, respectively) among the GICs.^[^ ^10^ ^]^ In subsequent years, ILBs were found to play an essential role in the superconductivity of these materials,^[^ ^11^, ^12^, ^13^, ^14^, ^15^ ^]^ which spurred efforts to identify nonnuclear‐bound electrons in materials other than GICs, leading to the discovery of such electrons in carbon nanotubes,^[^ ^16^, ^17^, ^18^ ^]^ fullerenes,^[^ ^19^ ^]^ MoS_2_,^[^ ^20^ ^]^ and MXenes.^[^ ^21^ ^]^

Around 2010, a new class of materials called electrides entered, and significantly activated, ILB research. An electride is by definition a stoichiometric compound in which electrons act as anions, i.e., a crystal where the lattice sites that one would expect to be occupied by an anion are empty and occupied instead by an electron (or electrons).^[^ ^22^ ^]^ Initiated by Dye in the 1970s, electride research was hampered for many years by the instability of electrides, mostly organic, under room temperature and ambient conditions.^[^ ^22^, ^23^ ^]^ A breakthrough came when an inorganic electride [Ca_24_Al_28_O_64_]^4+^(4e^−^) (C12A7:e^−^) was synthesized and found to be both thermally and chemically stable.^[^ ^24^ ^]^ C12A7:e^−^ is derived from mayenite [Ca_24_Al_28_O_64_]^4+^(O^2−^)_2_, having cages ≈4 Å in size in which oxygen ions are trapped loosely. ([Ca_24_Al_28_O_64_]^4+^ is the positively charged lattice framework, and (O^2−^)_2_ represents the oxygen ions trapped in the cage as counter anions.) Upon removing all the in‐cage oxygens, electrons enter the cages to maintain charge neutrality and form the C12A7:e^−^ electride. The electrons that have replaced the anions are called anionic electrons. (For a comprehensive review of electride research including their applications, see the previous study.^[^ ^25^ ^]^)

Electrides are classified, in general, by the dimensionality of the empty space accommodating the anionic electrons, which, in turn, determines the dimensionality of their electronic structures: C12A7:e^−^ may be considered a 0D electride, because anionic electrons are essentially trapped in the cages and are unable to move freely in any direction. Readers familiar with the F centers in alkali halides might want to imagine C12A7:e^−^ as a periodic array of F centers, namely, an alkali halide in which each halide ion is replaced by an electron. (To be precise, for electron concentrations beyond ≈1 × 10^21^ cm^−3^, the anionic electrons in C12A7:e^−^ are not rigidly confined in cages but can tunnel between the cages to become itinerant. It is, therefore, more appropriate to call C12A7:e^−^ a quasi‐0D electride.)

The discovery of this stable inorganic electride spurred the exploration for further inorganic electrides and their properties.^[^ ^25^ ^]^ Particular attention was paid to discover quasi‐2D electrides (Q2DEs) in which electrons accumulate in the gap (2D void) between cation layers and maintain overall charge neutrality. (A good summary of Q2DE research prior to 2017 is given in the previous study.^[^ ^26^ ^]^) The first Q2DE discovered was Ca_2_N, a metallic Q2DE in which the only band crossing *E*_F_ is an ILB, with anionic electrons confined in the interlayer space.^[^ ^27^ ^]^

The second Q2DE discovered was Y_2_C,^[^ ^28^ ^]^ featuring an electronic structure in strong contrast with that of Ca_2_N. Y_2_C is a semimetal, where an ILB and a Y 4*d* band hybridize in an intricate manner near *E*_F_ to exhibit Stoner‐type ferromagnetic fluctuations. Following the identification of Ca_2_N and Y_2_C as Q2DEs, a number of Q2DEs were found or proposed using the first‐principles density‐functional theory (DFT) calculations combined with databases and informatics.^[^ ^29^, ^30^, ^31^, ^32^, ^33^, ^34^ ^]^ Thus far, investigations of the properties of Q2DEs have focused on Ca_2_N and Y_2_C and have mostly been computational.

**Figure 1:**
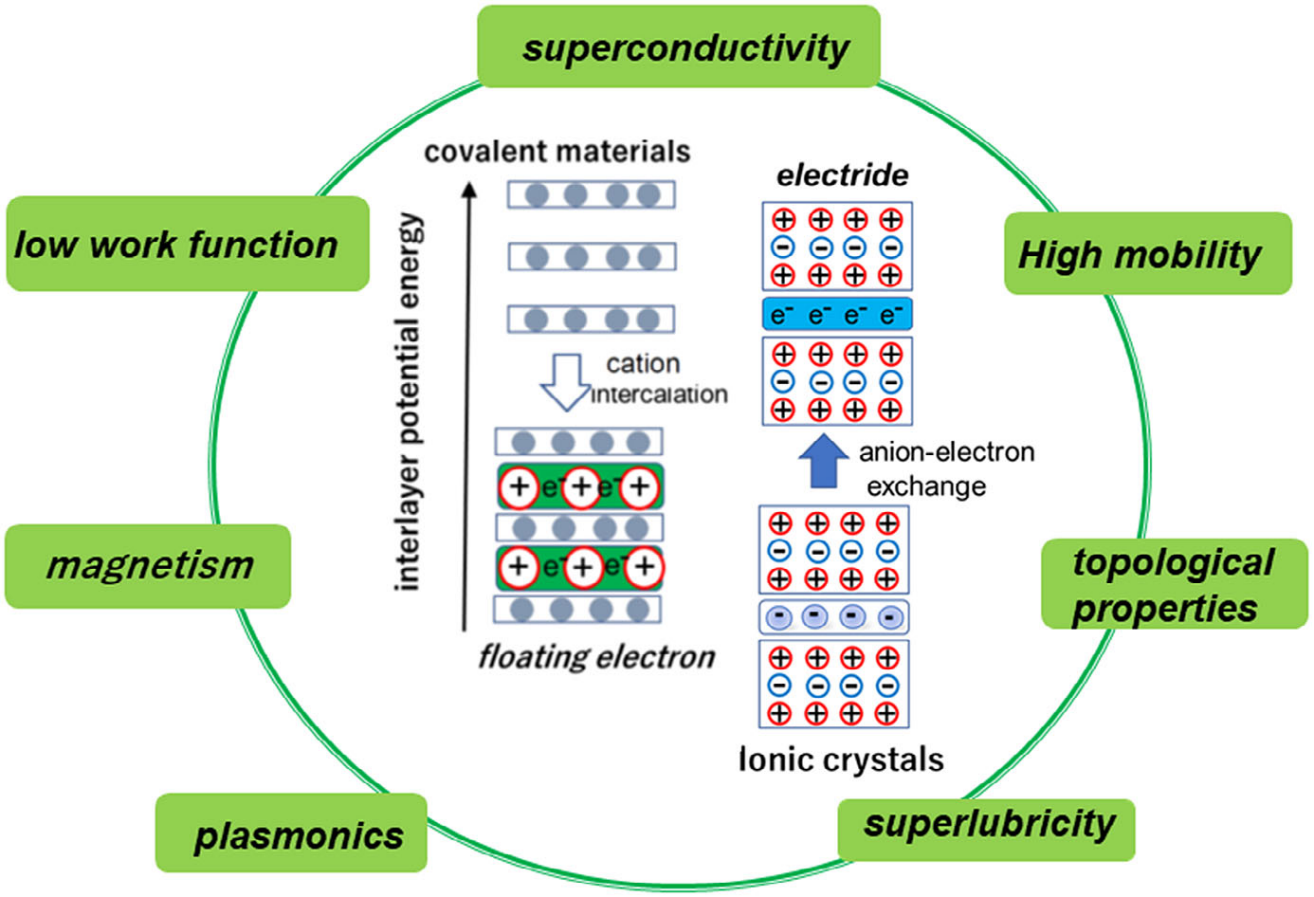
Two types of layered material hosting interlayer states near the Fermi level.

The nonnuclear floating electrons are induced in GICs and Q2DEs in contrasting ways, as shown in **Figure** **1**. To date, the research of these two types of materials has progressed independently in their respective communities. The aim of the present review is to bridge the gap and facilitate the exchange of information between the two communities.

## 2 Non‐Electrides

Graphite, the most stable allotrope of carbon under ambient conditions,^[^ ^35^, ^36^ ^]^ has been a target of intensive research in materials science since its accidental discovery by Acheson in the late 19th century.^[^ ^37^ ^]^ Graphite is a typical layered material in which over 99% of the cohesive energy is derived from C—C covalent bonds within a layer. Layers are bonded to each other by a weak dispersive force (van der Waals interaction), which allows graphite to be easily exfoliated mechanically to produce an atomically thin 2D crystal named graphene. The anisotropic structure of graphite also makes it possible to insert (intercalate) guest atoms or molecules between the layers to form stoichiometric intercalation compounds with increased layer–layer distance. Intercalation is driven by the energy gained from charge transfer between graphite and intercalants. Among the GICs, alkali metal and alkaline‐earth metal GICs are the most studied, because many of them become superconducting at very low temperatures.

### 2.1 Interlayer States in Graphite and Graphene

Graphite is a hexagonal crystal consisting of honeycomb layers of sp^2^‐hybridized carbon atoms with a C—C distance of 1.421 Å. In its standard form (Bernal graphite), the layers are stacked in an AB sequence with an interlayer distance of 3.328 Å, which is much larger than the C—C covalent bond length. As each honeycomb layer has two atoms in its unit cell, a unit cell of graphite contains four atoms, two in layer A and two in layer B.

**Figure 2:**
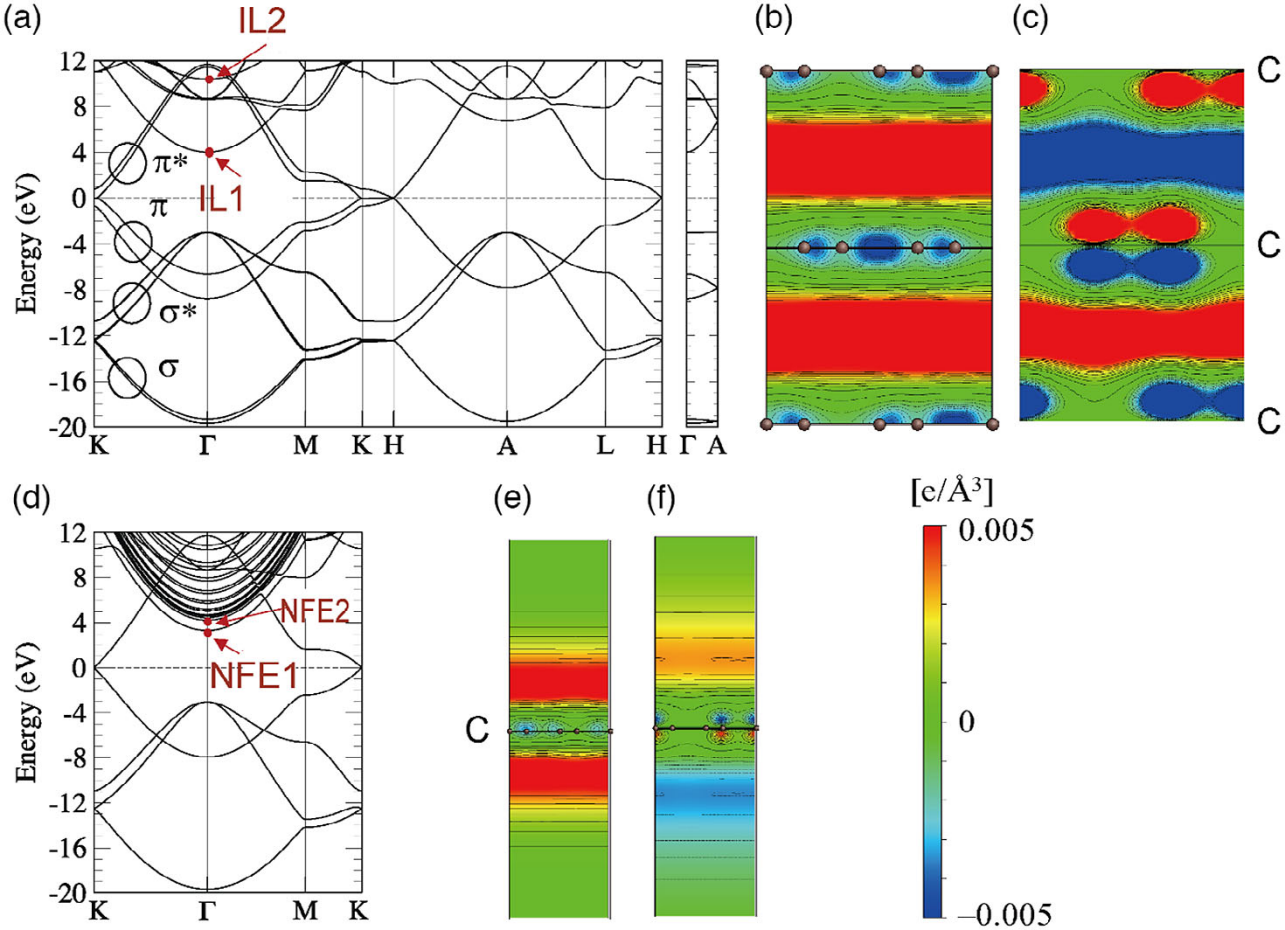
Electronic structures of a–c) graphite and d–f) MLG calculated by DFT in the local density approximation. The ILBs in (a) are labeled IL1 and IL2. The signed PEDs (square of the wave function multiplied by its sign) for IL1 and IL2 at Γ are shown in (b,c), respectively. Although MLG has no interlayer space, it has surface states similar to the interlayer states of graphite. These so‐called “NFE” states are labeled NFE1 and NFE2 in (d). The signed PED for NFE1 and NFE2 at Γ are shown in (e,f), respectively. Courtesy of Dr. Masayuki Toyoda.

In **Figure** **2**a, the electronic band structure of graphite calculated by local density approximation in the framework of DFT is shown. Here, the two lowest energy bands are the σ bonding states consisting of 2*s*, 2*p*_*x*_, and 2*p*_*y*_ orbitals of carbon. Both the σ bonding and σ* antibonding bands are fully occupied. The π bonding bands (π* antibonding bands), consisting of the C 2*p*_*z*_ orbitals, are fully occupied (unoccupied) except in the vicinity of K–H in the Brillouin zone. These π and π* bands are nearly degenerate along K–H, but there is a slight nondegeneracy, resulting from the finite orbital overlap between the layers. This nondegeneracy renders the system a semimetal with an equal number of electrons and holes, a finite density of states (DOS) *D*(*E*) at *E*_F_, and small Fermi surfaces around K–H. In a single‐layer graphene, the π and π* bands are strictly degenerate at K owing to the absence of interlayer overlap; hence, there are no carriers and *D*(*E*_F_) = 0.

In Figure 2a, the lowest unoccupied band near the Γ point, labeled IL1, has a free‐electron‐like parabolic dispersion in the *k*_*x*_–*k*_*y*_ plane. Figure 2b shows the plot of the signed partial electron density (PED), $\rho (r)=\text{sgn}(\varphi (r)){\left|\varphi \left(r\right)\right|}^{2}$, where *φ* is the electron wave function at Γ. This figure shows that an electron in this state has most of its probability amplitude (density) in the interlayer space. Such a band is referred to as an ILB.^[^ ^1^, ^5^, ^6^, ^38^ ^]^ (In recent literature, “NFE band” is often used to emphasize their parabolic in‐plane dispersion.) Interestingly, there is another parabolic band (labeled IL2) whose wave function at Γ has a node on the carbon plane (Figure 2c). The IL1 and IL2 bands correspond, respectively, to bonding and antibonding combinations of the two interlayer states associated with the two interlayer gaps in the unit cell of graphite.

Although monolayer graphene (MLG) has no interlayer space, it exhibits surface states very similar to the ILBs in graphite. As Figure 2d shows, these states (labeled NFE1 and NFE2) have parabolic dispersions. The plots of their wave functions (Figure 2e,f) show that the electrons in these states are virtually floating above the surface, separated by a distance of ≈1.7 Å from the carbon layer. It can be seen that NFE1 does not change sign through the inversion z→−z, whereas NFE2 does. This implies that NFE1 and NFE2 are, respectively, the bonding and antibonding combinations of the two surface states, one localized above the layer and the other localized below the layer. Such extra‐surface floating states are understood as image‐potential states associated with the graphene layer.^[^ ^39^ ^]^

### 2.2 Graphite Intercalation Compounds

Although graphite can accommodate both donors and acceptors in its interlayer space, donor‐intercalated GICs are of interest in terms of the ILB states, because the donors are ionized to have a positive charge in the interlayer space and attract the electrons. This lowers the energy of the ILB, leading to its occupation.

**Figure 3:**
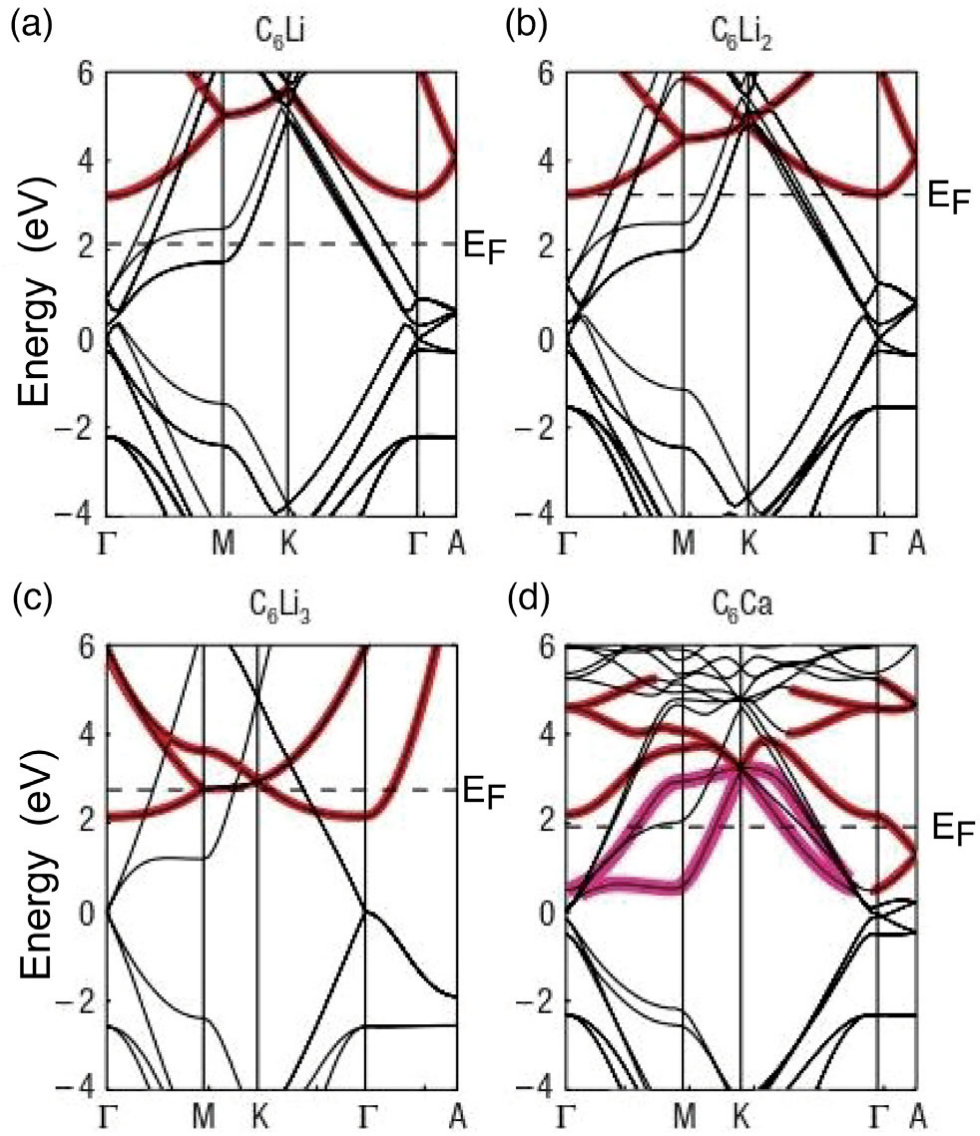
Band structures of a) C_6_Li, b) C_3_Li, c) C_2_Li, and d) C_6_Ca calculated using DFT. The ILBs are highlighted in red. The Fermi level is indicated by dashed lines. Adapted with permission.^[^ ^11^ ^]^ Copyright 2005, Springer Nature.

**Figure** **3** shows the electronic band structures of Li‐ and Ca‐intercalated GICs with the ILBs highlighted in red.^[^ ^11^ ^]^ Figure 3a–c, depicting the band structures of Li GICs with increasing Li concentration, shows how the ILB, which is above *E*_F_ in C_6_Li, drops in energy to be filled partially by electrons in C_6_Li_3_. Among these three Li GICs, only C_6_Li_3_ (C_2_Li) becomes superconducting below *T*_c_ = 1.9 K.^[^ ^40^ ^]^

By comparing Figure 2 and 3, it is clear that the ILBs in Li GICs are lower in energy than the IL1 band of graphite, implying that the attractive potential of Li ions plays a dominant role in lowering the energy of the GIC bands, although the increase in the interlayer distance and the hybridization of the ILB with the Li 2*s* states also contribute.

In the case of C_6_Ca, which is isostructural with C_6_Li, the number of valence electrons is identical to that of C_6_Li_2_, but the hybridization effect is much more pronounced than in Li GICs. Hence, the band structure of C_6_Ca differs significantly from that of graphite, especially near *E*_F_ where the bands have a hybrid atomic‐interlayer character.

**Figure 4:**
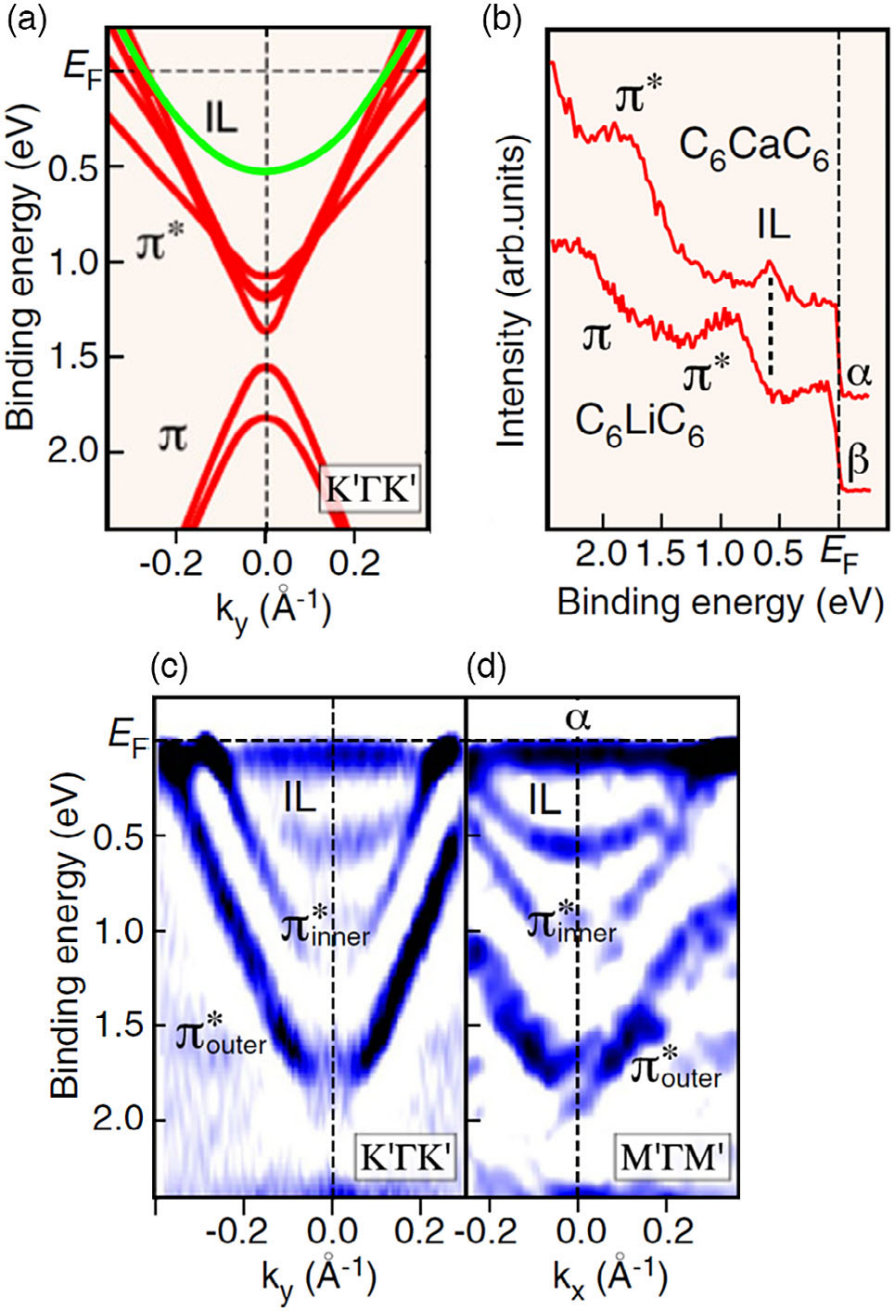
a) Calculated energy band structure of bilayer graphene intercalated with Ca (C_6_CaC_6_). b) ARPES spectra of C_6_CaC_6_ and C_6_LiC_6_ at the Γ point. c,d) Band dispersion of C_6_CaC_6_ obtained by ARPES measurements along the high symmetry lines K′ΓK′ and M′ΓM′, respectively, showing ILBs (indicated as IL). Adapted with permission.^[^ ^41^ ^]^ Copyright 2012, The National Academy of Sciences.

MLG is the thinnest system produced by the exfoliation of graphite. Similarly, the thinnest GIC is bilayer graphene intercalated between the two carbon layers.^[^ ^41^ ^]^ Such atomically thin GICs (C_6_CaC_6_ and C_6_LiC_6_) were fabricated on a SiC substrate, and their electronic band structures were examined by angle‐resolved photoemission spectroscopy (ARPES). **Figure** **4**a,c shows, respectively, the calculated and measured band dispersions along the K′ΓK′ line in the Brillouin zone. The parabolic ILB (indicated as IL) is clearly observed in both. (There are two π* bands in Figure 4c,d, because the substrate makes the two carbon layers inequivalent.) The measured dispersions along K′ΓK′ (Figure 4c) and M′ΓM′ (Figure 4d) are similar, indicating the isotropic nature of the ILB. Such an ILB is present in C_6_CaC_6_ but not in C_6_LiC_6_, as shown in the spectrum at Γ in Figure 4b.

### 2.3 Superconductivity of GICs

Among the GICs, superconductivity was first observed in C_8_K.^[^ ^42^ ^]^ Despite the low transition temperature (0.15 K), this observation caught the eyes of researchers and led to the discovery of a number of superconducting GICs.^[^ ^43^ ^]^ These compounds are donor GICs with alkali or alkaline‐earth metals as intercalants. As donor intercalation lowers the ILB toward *E*_F_, the question centered on whether it was the electrons in the carbon π* band or those in the ILB that were responsible for the superconductivity. Csányi and co‐workers pointed out that there is a clear coincidence between the ILB occupation and the occurrence of superconductivity. This is shown for the case of Li GICs (C_6_Li_3_, C_6_Li_2_, and C_6_Li) in Figure 3. As the Li concentration increases, the ILB drops and is finally occupied in C_6_Li_3_, which is the only superconducting compound (*T*_c_ = 1.9 K) in this group.

The observation of superconductivity in C_6_Ca with *T*_c_ = 11.5 K (the highest transition temperature among the GICs reported to date),^[^ ^10^ ^]^ which increases to 15.1 K under a pressure of 7.5 GPa,^[^ ^44^ ^]^ garnered wider interest in superconducting GICs: The 100‐fold increase in *T*_c_ going from K to Ca invigorated efforts to uncover the underlying mechanism, particularly the role of the ILBs.

An ARPES measurement by Sugawara and his colleagues revealed a superconducting gap of 1.8–2.0 meV on the Fermi surface derived from the ILB, whereas the gap on the Fermi surface of the π* band was almost undetectable (0.2 ± 0.2 eV).^[^ ^14^ ^]^ A later ARPES study by Yang et al. found superconducting gaps on the Fermi surfaces corresponding to both the ILB and the π* band, induced by their coupling with the out‐of‐plane phonon modes of carbon.^[^ ^15^ ^]^ More significantly, they found that the interaction between the two bands mediated by these phonon modes makes a substantial contribution to the total electron–phonon coupling, meaning that the occupation of the ILB is necessary but not sufficient; ILB‐π* interactions via phonons are also required. This is consistent with the theoretical finding by Boeri et al.^[^ ^13^ ^]^ that the interband phonon scattering between the ILB and the π* band is crucial.

There is a general consensus today that the superconductivity of these compounds results from a Bardeen–Cooper–schrieffer (BCS)‐type *s*‐wave pairing mediated by phonons for which electrons in both the π* band and the ILB contribute. The dimensionless electron–phonon coupling parameter *λ* was determined to be 0.70 from a specific heat measurement.^[^ ^45^ ^]^ Concerning the question of how to achieve a higher *T*_c_ in GICs, there are some hints in the literature: the ternary intercalation compound C_8_KH_*x*_ is known to have a higher *T*_c_ (=0.22 K for *x* = 0.19) than C_8_K.^[^ ^46^, ^47^ ^]^ A theory based on a simplified model suggests that the co‐intercalation of a large‐valence element and a heavy element together with a large effective mass of the electrons will increase *T*_c_.^[^ ^48^ ^]^ Also, the partial replacement of C with B in Li GICs is predicted to boost *T*_c_.^[^ ^49^ ^]^

### 2.4 Interlayer (NFE) States in Other Layered Materials

**Figure 5:**
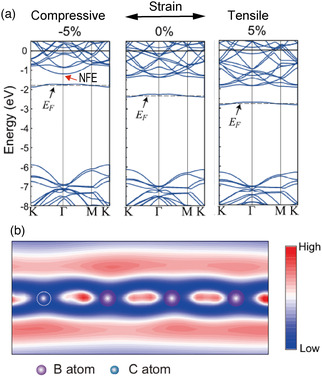
Electronic structure of an h‐BN monolayer under strain containing C impurities (donor) substituting B. The calculation was performed for a 4×4 supercell using DFT in the local‐density approximation. a) Band structure with/without strain. b) Electron density corresponding to the conduction band minimum at the Γ point under 5% compressive strain. Adapted with permission.^[^ ^50^ ^]^ Copyright 2016, The American Physical Society.

Other than the materials discussed earlier, the emergence of ILBs has been predicted for many layered materials, including h‐BN,^[^ ^4^ ^]^ MoS_2_,^[^ ^20^ ^]^ and MXenes.^[^ ^21^ ^]^ An interesting system is monolayer h‐BN.^[^ ^4^ ^]^ Although h‐BN is made of honeycomb layers similar to graphene, there is a fundamental gap above the valence band edge, making the system a wide‐gap semiconductor. This gives us the expectation that the ILB at Γ may be the bottom of the conduction band. A DFT calculation shows that this is the case if it is compressed by 5% (**Figure** **5**).^[^ ^50^ ^]^ This finding suggests that an electron‐doped h‐BN monolayer will show strain‐dependent electronic transport properties.

Atomic sheets can be rolled up into 1D nanotubes. Carbon nanotubes^[^ ^16^, ^17^, ^18^ ^]^ and BN nanotubes^[^ ^51^, ^52^, ^53^ ^]^ are, therefore, expected to exhibit a floating NFE state having their wave function amplitudes both inside and outside of the tube wall. As regards “0D layered materials,” intra‐cage states are known to exist in C_60_, C_70_, and larger fullerenes.^[^ ^19^ ^]^ In solid C_60_, inter‐cage bands coupled with intra‐cage states are also predicted to occur: both the intra‐cage states and inter‐cage bands are downshifted when Sr is incorporated into C_60_ to form Sr_6_C_60_.^[^ ^54^ ^]^

## 3 (Quasi‐)2D Electrides: Crystal Structures and Electronic States

### 3.1 Bulk Materials

The electronic structure and properties of electrides depend critically on the dimensionality of the void that confines the anionic electrons. Subsequent to the discovery of the quasi‐0D electride C12A7:e^−^, dozens of layered materials were identified as Q2DEs in which anionic electrons are confined loosely in the interlayer space.

Alkaline‐earth subnitrides M_2_N (M = Ca, Sr, and Ba) are layered materials known since the 1950s. In 2000, Gregory et al. analyzed their structures and pointed out the possibility of there being excess electrons in the interlayer space rather than bound in atomic orbitals.^[^ ^55^ ^]^ Subsequently, Lee et al. showed by DFT calculation that Ca_2_N is a Q2DE and went on to confirm experimentally the presence of Q2D electrons.^[^ ^27^ ^]^ This work was followed by a DFT calculation showing that Sr_2_N and Ba_2_N are also Q2DEs.^[^ ^56^ ^]^ The three subnitrides share the same anti‐CdCl_2_ structure (space group $R\bar{3}m$) and have very similar band structures.

**Figure 6:**
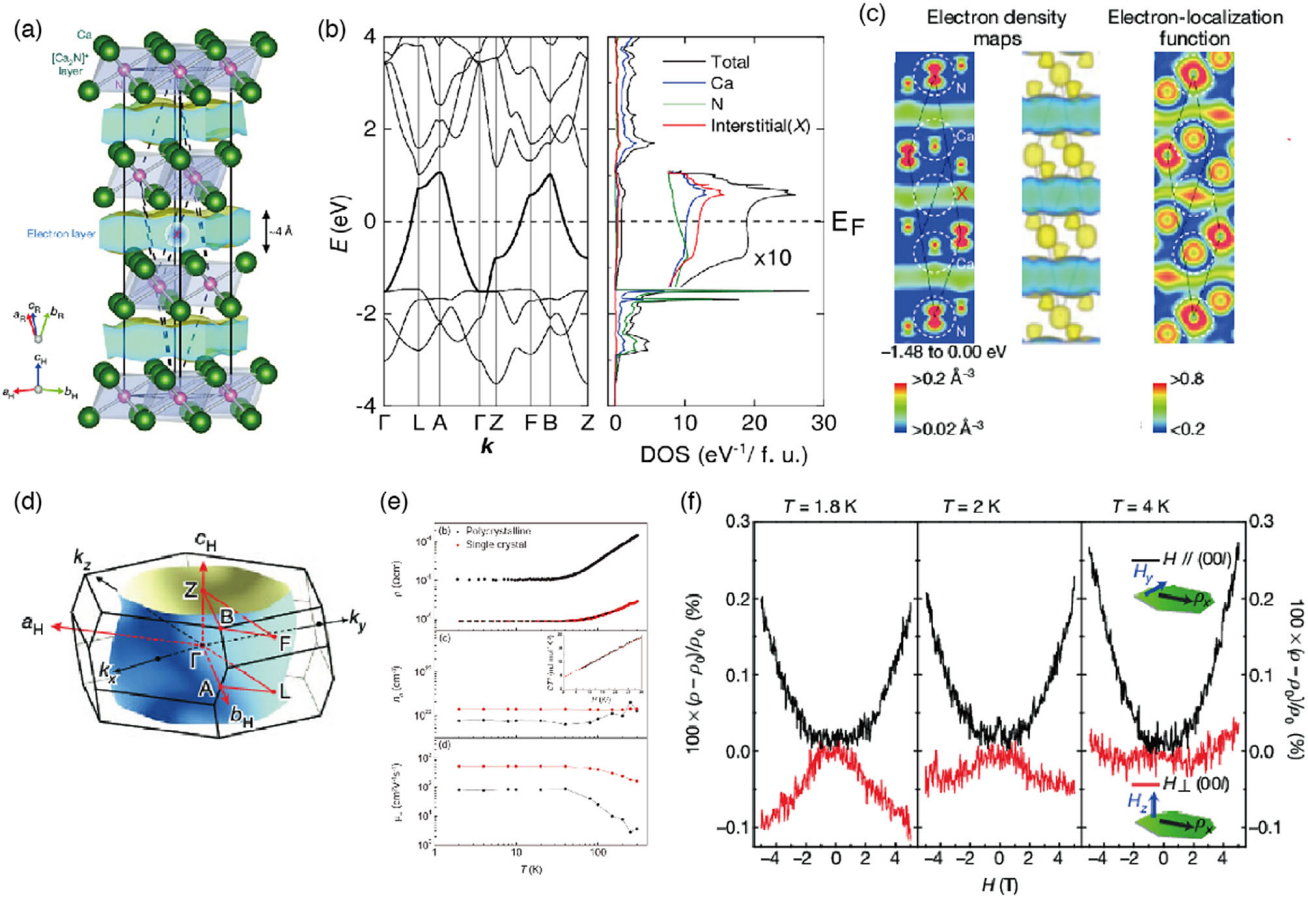
a) Crystal structure of Ca_2_N. b–d) Electronic structure of Ca_2_N calculated using DFT. *E*_F_ is set to zero. b) Energy band structure (left) and DOS (right). c) PED (left) and its isosurface (center) for the ILB (energy within 1.48 eV below *E*_F_), and electron localization function (right). d) Fermi surface. e,f) Measured electron transport properties of Ca_2_N: e) temperature dependence of resistivity (upper panel), electron concentration (middle panel), and Hall mobility (bottom panel) for both single‐crystal and polycrystal samples. f) Magnetoresistance measured at different temperatures for a single‐crystal sample. Adapted with permission.^[^ ^27^ ^]^ Copyright 2013, Springer Nature.

As shown in **Figure** **6**a, each layer of Ca_2_N is hexagonal and made of edge‐sharing NCa_6_ octahedra. A (conventional) unit cell consists of three layers, each being a Ca/N/Ca trilayer, piled on top of each other in an ABC stacking sequence. Hereafter, the Ca/N/Ca trilayer will be referred to as a layer. The interlayer separation of 3.81 Å is larger than that of van der Waals materials (e.g., 3.33 Å for graphite). The distance between Ca and N (2.44 Å) is nearly equal to the sum of the ionic radii of Ca^2+^ (1.0 Å) and N^3−^ (1.46 Å), implying that the Ca_2_N layer is cationic with net charge +1 and can be written as [(Ca^2+^)_2_N^3−^]^+^. This positive charge is compensated for by electrons in the interlayer space, resulting in a charge‐neutral crystal in the form [Ca_2_N]^+^·e^−^. This expectation is substantiated by the DFT band structure of Figure 6b, showing that there is only one conduction band (bold line) around *E*_F_ and that this band is half‐filled. Near *E*_F_, the band has a free‐electron‐like dispersion in the *ab* plane, whereas its dispersion in the *c*‐direction (perpendicular to the layer plane) is small, implying that it is a quasi‐2D band with strong anisotropy. This anisotropy is consistent with the Fermi surface, which is a nearly perfect cylinder centered at the Γ point in the first Brillouin zone (Figure 6d). The density of the ILB electrons (left and middle panels of Figure 6c) indicates that they occupy the interlayer space with considerable uniformity, confirming the ILB character. (Note, however, that they are hybridized to some extent with the atomic orbitals of N and Ca.) The interlayer electrons in Ca_2_N are tightly bound in the *z*‐direction owing to the large thickness of the ionic layers [Ca_2_N]^+^, leading to a small dispersion in the *z*‐direction. This is in contrast to the ILBs in graphite, which are considerably more isotropic. Figure 6c suggests that the anionic electrons are loosely bound in non‐atomic *s*‐like orbitals centered at the interstitial sites (Wyckoff position *b*), marked by X in the left panel of Figure 6c, forming interstitial quasi‐atoms. The electron localization function (ELF; right panel of Figure 6c) indicates unshared‐electron interaction (physical bonding as opposed to chemical bonding) between the interstitial electrons at X and the [Ca_2_N]^+^ layer (white dashed circles).

The valence bands below the ILB (*E*‐*E*_F_ < −1.5 eV) consist mainly of N 2*p* orbitals, whereas the unoccupied bands above the ILB (*E*‐*E*_F_ > 1.5 eV) derive from the Ca 3*d* orbitals. The Fermi energy is in the middle of these two types of bands and separated from either, which tends to suppress the hybridization of the ILB with other bands near *E*_F_.

The number of ILB electrons estimated by integrating the number of occupied states is 1.0 per (primitive) unit cell, consistent with the chemical formula [Ca_2_N]^+^·e^−^. From this, the conduction electron density can be determined to be 1.37 × 10^22^ cm^−3^, which is in remarkably good agreement with the carrier density (*n* type) obtained from a Hall measurement of 1.39 × 10^22^ cm^−3^ at 300 K. The carrier concentration is nearly constant down to 2 K (Figure 6e). The in‐plane electron mass obtained from the Fermi surface information is 1.8–2.2 *m*_0_ (*m*_0_ is the bare electron mass), which compares well with the values of 1.9 *m*_0_ from a heat capacity measurement and 2.5 *m*_0_ from the plasma frequency.^[^ ^27^ ^]^ The Hall measurement revealed the mobility to be 160 cm^2^V^−1^s^−1^ at 300 K (520 cm^2^ V^−1^s^−1^ at 2 K), which is considerably higher than those of NFE metals such as Na (several tens of cm^2^V^−1^s^−1^). The resistivity is proportional to *T* ^2^ below 120 K, suggesting that the dominant scattering mechanism is electron–electron interaction, and that the scattering by phonons is weak. The low‐temperature magnetoresistance is highly anisotropic and negative (positive) for out‐of‐plane (in‐plane) magnetic fields (Figure 6f), which may be understood from the 2D character of the conduction electrons. Further support for the calculated band structure comes from an ARPES measurement, which indicates that the band dispersion and Fermi surface of the ILB agree with the calculation if the calculated energy bands are shifted upward by 0.19 eV.^[^ ^57^ ^]^ (This shift is ascribed to the depletion of the anionic electrons near the cleaved surface.)

Subsequent to the discovery of Ca_2_N, Inoshita et al. conducted a systematic search for Q2DEs, combining DFT calculations and database screening, based on a working hypothesis that 1) the material is a layered binary compound with interlayer separation >3.0 Å, and 2) there is an excess of electrons as indicated by the sum of the standard oxidation numbers of the constituent ions >0.^[^ ^28^ ^]^ The search identified rare‐earth subcarbides Y_2_C and *Ln*_2_C (*Ln *= Gd, Tb, Dy, Ho, and Er) as Q2DEs. All these materials share the same anti‐CdCl_2_ crystal structure as Ca_2_N. *Ln*_2_C is ferromagnetic with Curie temperatures in the range of 100–351 K. An important difference between these carbides and the nitrides is the number of excess electrons. While the net ionic charge per formula unit (f.u.) of Ca_2_N is +1, that for the carbides is +2 (assuming the standard oxidation numbers for the cation *M* and carbon of +3 and −4, respectively). This implies that a primitive unit cell of a carbide contains two anionic electrons, and its chemical formula can be written as [*M*_2_C]^+2^·2e^−^. The *c* lattice constant of Y_2_C is 17.96 Å, which is considerably smaller than the value of 19.10 Å for Ca_2_N. This lattice shrinkage, which is seen in *Ln*_2_C as well, can be explained by the increased Coulomb attraction between the metal ions and the interlayer anionic electrons.

**Figure 7:**
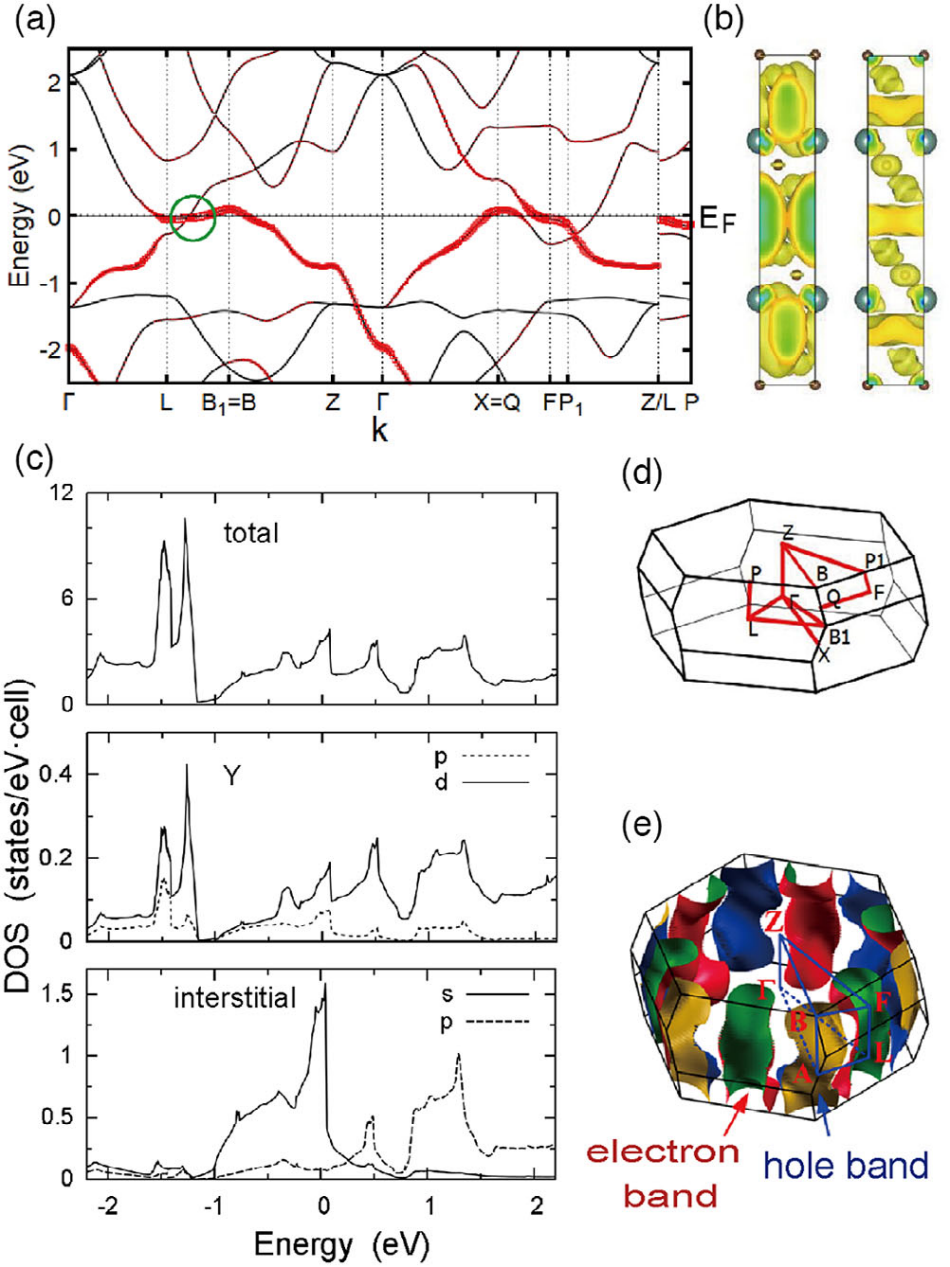
Electronic structure of Y_2_C in the nonmagnetic phase calculated using DFT. a) Band structure, b) PED isosurface, c) DOS, d) Brillouin zone, and e) Fermi surface. The left (right) panel of (b) corresponds to electron energy −0.05 eV < *E*‐*E*_F_ < 0.05 eV (−0.35 eV < *E*‐*E*_F_ < −0.25 eV). The circle in (a) denotes the band crossing that renders the system a Dirac nodal‐line semimetal. The DOS in the bottom panel of (c) (indicated as “interstitial“) is obtained by placing an empty sphere of 1.8 Å radius at the interstitial sites (marked as X in Figure 6c) and projecting the wave functions onto this sphere. b) Reproduced under the terms of the CC‐BY 3.0 license.^[^ ^28^ ^]^ Copyright 2014, The Authors, Published by The American Physical Society. c) Reproduced with permission.^[^ ^59^ ^]^ Copyright 2015, The American Physical Society. e) Reproduced with permission.^[^ ^58^ ^]^ Copyright 2014, The American Chemical Society.

With two excess electrons in the primitive unit cell, one would expect Y_2_C to be a band insulator. DFT calculation shows this naive expectation to be incorrect, as can be seen from the band structure shown in **Figure** **7**a: Y_2_C is a semimetal with two conduction bands overlapping near *E*_F_. (In this figure, the ILBs are highlighted in red using a fat band scheme; i.e., the widths indicate the interlayer weight.) The band structure combined with the partial DOS (PDOS) (Figure 7c) indicates that the electronic states in the range −1 to 0.2 eV are mainly of ILB character, with which a broad Y *d* band having its bottom slightly below *E*_F_ overlaps and hybridizes in a subtle manner to form a two‐band system. Reflecting this semimetallicity, the Fermi surface has two types of pocket, electron‐like and hole‐like, both of which are rugged cylinders in shape (Figure 7e). The electronic structure is less anisotropic than that of Ca_2_N. The density of carriers estimated from the volume of the Fermi surface is 0.19/f.u. for both electrons and holes.^[^ ^58^ ^]^ The effective masses obtained from the calculated DOS at the Fermi level assuming the free electron model are 5.1 *m*_0_ (electrons) and 8.4 *m*_0_ (holes).^[^ ^58^ ^]^ Compared with Ca_2_N, the in‐plane distribution of anionic electrons is less uniform and more localized in both the out‐of‐plane and in‐plane directions, as shown by the PED plots in Figure 7b.^[^ ^28^, ^59^, ^60^ ^]^

The electronic structure calculation prompted Zhang et al.^[^ ^58^ ^]^ to synthesize and characterize polycrystalline samples. The *D*(*E*_F_) obtained from the specific heat was 3.95 states/eV·f.u., in reasonably good agreement with the calculated value of 3.27 states/eV·f.u. The resistivity has a metallic dependence on temperature and is equal to 217 μΩ·cm at 300 K. The Hall coefficient is negative and strongly dependent on temperature. The number of carriers assuming a single free‐electron band is 4.37 × 10^21^ cm^−3^, which is far smaller than the value of ≈2.94 × 10^22^ cm^−3^ obtained from the formula [M_2_N]^+2^·2e^−^.^[^ ^58^ ^]^ These results are consistent with the calculated semimetallic band structure. The dispersion and Fermi surface obtained from ARPES measurements by Horiba et al. are in good agreement with the calculation.^[^ ^61^ ^]^

**Figure 8:**
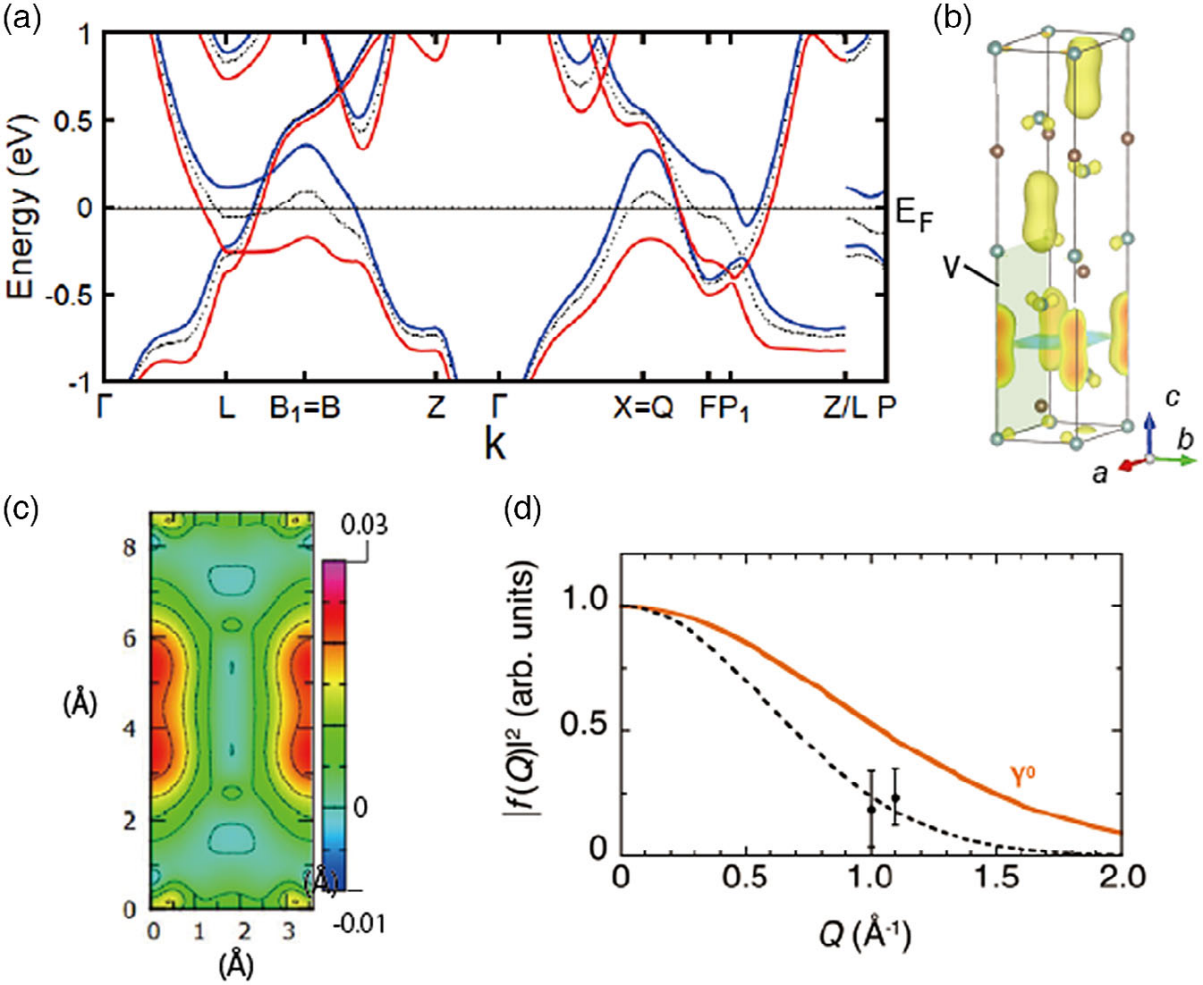
a–c) Electronic structure of Y_2_C in the ferromagnetic phase calculated using DFT. a) Band structure, with the red and blue lines indicating the majority‐spin and minority‐spin bands, respectively. For comparison, the energy bands in the nonmagnetic phase are plotted by black dotted lines. b) Magnetization isosurface. c) Contour plot of magnetization in the *ac* plane indicated by V in (b). Reproduced with permission.^[^ ^59^ ^]^ Copyright 2015, The American Physical Society. d) Squared magnetic form factor of Y_2_C derived from an inelastic neutron scattering measurement.^[^ ^68^ ^]^ The orange line shows the squared form factor calculated from the magnetization density of a 4*d* electron in an yttrium atom. The latter cannot explain the measurement result. The dashed line is a guide for the eye. d) A modified version of Figure 3 in the previous study^[^ ^68^ ^]^ provided by Dr. Hiromu Tamatsukuri.

We have, thus far, discussed the electronic structure of Y_2_C, assuming that it is nonmagnetic. DFT calculation actually shows that its ground state is weakly ferromagnetic.^[^ ^59^ ^]^ When spin polarization is considered, each of the two conduction bands splits into two (red and blue curves in **Figure** **8**a), producing a small magnetic moment of 0.33–0.38 μ_B_/f.u. (depending on the calculation method).

Figure 8b,c shows the isosurface of the magnetization density and its contour plot in the *ac* plane (indicated as V in Figure 8b), respectively. Remarkably, the bulk of the magnetization is localized around the interstitial sites in a peanut shape instead of at Y sites, which are at the corners of Figure 8c. The contribution of Y to the magnetization density, estimated from the ionic radius of 0.9 Å for Y^3+^, accounts for only 7.1% of the total magnetization, implying that the ferromagnetism is induced by the anionic nonnuclear electrons. Such Stoner‐type ferromagnetism originating from the spin polarization of anionic electrons has been predicted to occur in YCl,^[^ ^62^ ^]^ LaBr_2_,^[^ ^63^ ^]^ La_2_Br_5_,^[^ ^63^ ^]^ and Ca_5_Ga_2_N_4_^[^ ^64^ ^]^ at ambient pressure and in alkali metals under ultrahigh pressures.^[^ ^65^ ^]^

Turning to experiments, several research groups have reported magnetic measurements of Y_2_C using different polycrystal and single‐crystal samples.^[^ ^58^, ^60^, ^66^, ^67^ ^]^ The results are sample‐dependent, but no magnetic transition has been observed, thus far, down to sub‐liquid‐He temperatures. The temperature dependence of magnetization, in general, follows the Curie–Weiss law ≈ (*T*‐Θ)^−1^ with Weiss temperature Θ < 0 except possibly at *T* < 20 K. (The magnetization reported for a single crystal in the previous study,^[^ ^66^ ^]^ however, shows almost no temperature dependence and is Pauli‐like.) The magnetic moment is strongly sample‐dependent, ranging from 0.085^[^ ^66^ ^]^ to 2.82 μ_B_/f.u.^−1^,^[^ ^60^ ^]^ and increases sharply, as the temperature decreases below 20 K.^[^ ^58^, ^66^ ^]^

The in‐plane resistivity of Y_2_C was found to be metallic, decreasing monotonically as *T* decreases to 15 K and then starting to increase as ln *T* as *T* decreases further.^[^ ^60^ ^]^ This increase in resistivity at low temperatures was ascribed to the existence of magnetic moments. The in‐plane magnetoresistance is strongly anisotropic as to the direction of the magnetic field but always negative, which is explained by the suppression of ferromagnetic fluctuations by the magnetic field.^[^ ^60^ ^]^

Tamatsukuri et al. conducted a neutron inelastic scattering measurement on polycrystalline Y_2_C and observed magnetic scattering induced by ferromagnetic fluctuations near the momentum transfer *Q* ≈ 0.^[^ ^68^ ^]^ The dependence of the complex susceptibility on *Q* and *E* (energy transfer) shows that Y_2_C is in a paramagnetic state near a ferromagnetic instability. As *Q* increases, the magnetic form factor *f*(*Q*) decays to 0 much more rapidly than *f*(*Q*) for the 4*d* state of yttrium (Figure 8d), from which the spatial extent of the magnetic moments is estimated to be twice that of Y 4*d*. This excludes the possibility of yttrium‐induced magnetism and suggests the anionic electrons to be the main contributor to the magnetism, consistent with the calculation. Also, Tamatsukuri interpreted the negative Weiss temperature observed in terms of the mode‐coupling theory of ferromagnetic fluctuations.^[^ ^69^, ^70^, ^71^ ^]^

Hiraishi et al. reported muon spin rotation/relaxation measurements and argued that the Curie–Weiss‐like behavior of the susceptibility in polycrystalline samples is intrinsic, not due to impurities.^[^ ^66^ ^]^ They assumed that the magnetic fluctuations originate from the spin polarization of Y 4*d*, but the result can also be interpreted as arising from magnetic moments localized around interstitial sites.

The reason for the strong sample dependence of the magnetism of Y_2_C is not yet clear. However, as a Y *d*‐like band and an ILB hybridize near *E*_F_, the magnetism is expected to be sensitive to the position of *E*_F_ relative to these bands. It would, therefore, be possible that impurities or slight nonstoichiometry shifts *E*_F_, which, in turn, affects the magnetism.

**Figure 9:**
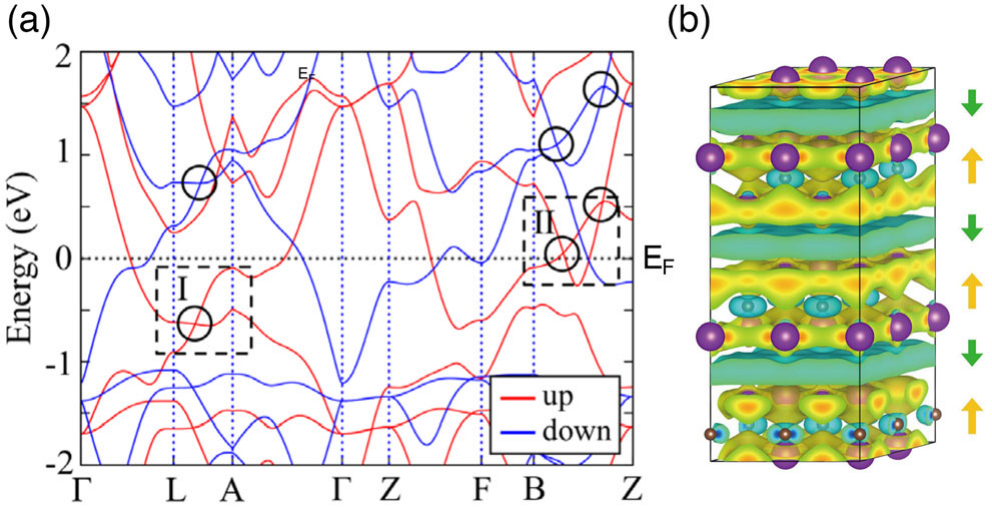
a) Energy band structure of Gd_2_C calculated using DFT. The red and blue curves denote the majority‐spin and minority‐spin bands, respectively. The circles indicate band crossings that render the system a Weyl nodal‐line semimetal. Reproduced with permission.^[^ ^123^ ^]^ Copyright 2020, The American Physical Society. b) Magnetization density of anionic electrons with energy within 0.1 eV below *E*_F_. Reproduced under the terms of the CC‐BY 3.0 license.^[^ ^28^ ^]^ Copyright 2014, The Authors, Published by The American Physical Society.

*Ln*_2_C (*Ln *= Gd, Tb, Dy, and Ho) is known to exhibit ferromagnetism derived from the *f* electrons of *Ln* ^+3^ ions with the Curie temperatures of 351 K (Gd_2_C), 266 K (Tb_2_C), 168 K (Dy_2_C), and 100 K (Ho_2_C).^[^ ^72^, ^73^, ^74^, ^75^ ^]^ (No magnetic measurement has been reported for Er_2_C.) DFT calculation revealed that these materials are Q2DEs with very similar band structures.^[^ ^28^ ^]^ The calculated magnetic moments for Gd_2_C, 15.46 μ_B_/f.u.^[^ ^28^ ^]^ and 14.68 μ_B_/f.u.,^[^ ^72^ ^]^ are in fairly good agreement with the experimental values of 15.6 μ_B_/f.u.^[^ ^76^ ^]^ and 14.52 μ_B_/f.u.^[^ ^72^ ^]^ The calculated band structure shown in **Figure** **9**a resembles that of spin‐polarized Y_2_C (Figure 8a) except that the exchange splitting is larger for Gd_2_C. Near *E*_F_, the majority‐spin (up) band is of Gd 4*d* character with electrons localized in the Gd layer, whereas the minority‐spin (down) band is an ILB with electrons confined in the interlayer space. As a result, the spin density of the anionic electrons shows a stripe pattern with up and down spins alternating in the *c*‐direction (Figure 9b). In the previous study,^[^ ^76^ ^]^ the ferromagnetism of Gd_2_C was ascribed to the Ruderman–Kittel–Kasuya–Yosida (RKKY) interactions^[^ ^77^ ^]^ between the Gd 4*d* electrons mediated by the ILB electrons.

The measured resistivity of Gd_2_C is highly anisotropic in the range 40 K < *T* < 350 K, the out‐of‐plane component being approximately six times larger than the in‐plane component, and scales as *T* ^*n*^ (0.8 < *n* < 1.5).^[^ ^76^ ^]^ The magnetoresistance also shows strong anisotropy as to the magnetic field direction. The conduction electron density obtained from a Hall measurement of 2.9 × 10^22^ cm^−3^ is in excellent agreement with the value of 2.84 × 10^22^ cm^−3^ estimated from the formula [Gd_2_C]^+2^·2e^−^.

**Figure 10:**
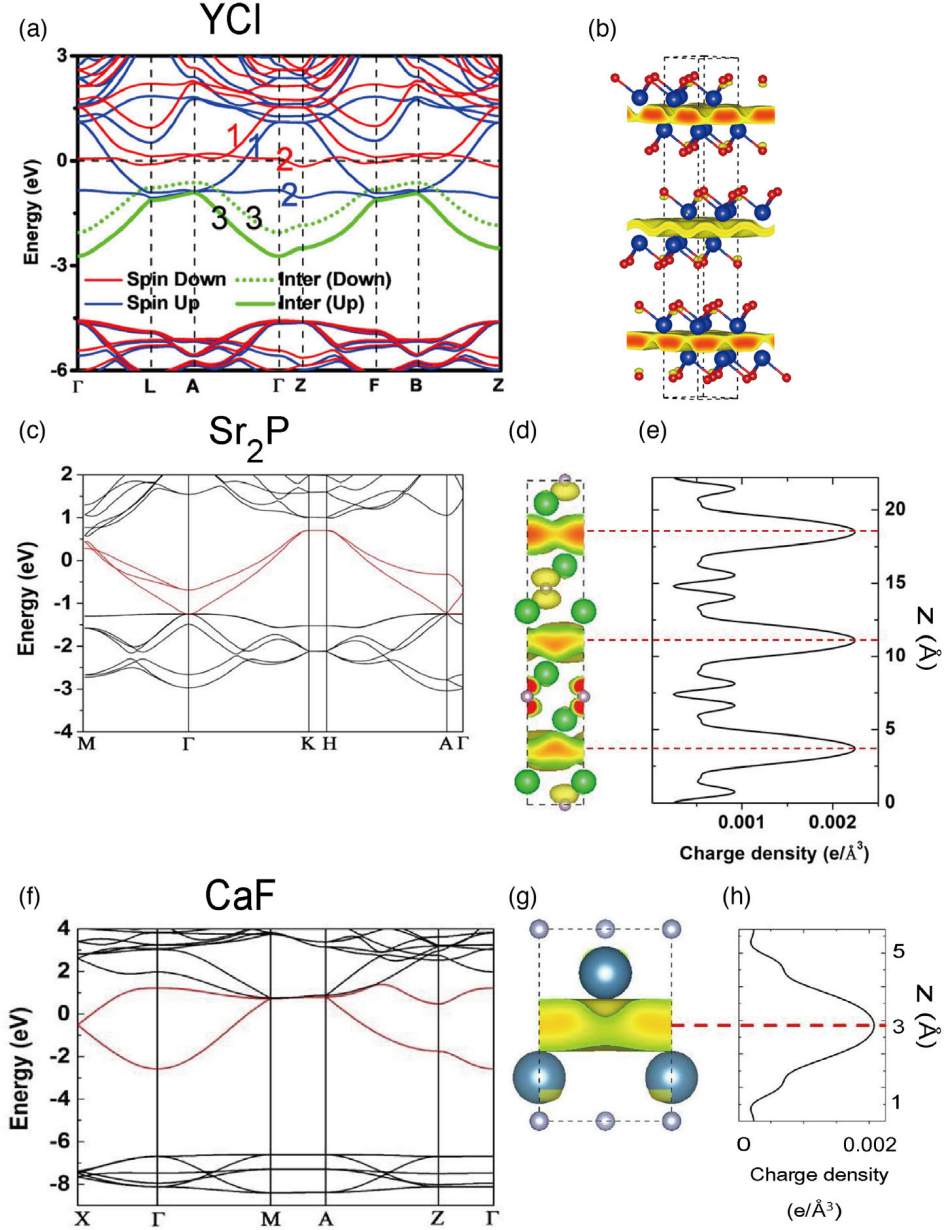
a,b) Electronic structure of YCl in the ferromagnetic phase calculated using DFT. a) Band structure with the ILBs marked in green. b) The PED isosurface of the ILBs. a,b) Reproduced under the terms of the CC‐BY 4.0 license.^[^ ^62^ ^]^ Copyright 2018, The Author, Published by Springer Nature. c–e) Electronic structure of Sr2P calculated using DFT. c) Electronic band structure. d) PED isosurface and e) plane‐averaged PED. f–h) Electronic structure of CaF calculated using DFT. f) Electronic band structure, g) PED isosurface, and h) plane‐averaged PED. The PED in (d,e) and (g,h) corresponds to the electron energy within 0.1 eV below *E*_F._ c–h) Reproduced with permission.^[^ ^30^ ^]^ Copyright 2016, The American Chemical Society.

*M*Cl (*M* = Y and Sc) has the ZrCl structure (space group $R\bar{3}m$), in which close‐packed quadruple layers Cl–*M*–*M*–Cl are stacked. DFT calculation shows these compounds to be ferromagnetic with magnetizations 1.58 μ_B_/f.u. (YCl) and 1.53 μ_B_/f.u. (ScCl).^[^ ^62^ ^]^ In the spin‐polarized band structure of YCl shown in **Figure** **10**a, the ILBs are indicated as “3.” The difference in electronic structure between YCl and Y_2_C may be understood from their difference in a crystal structure. A primitive unit cell of YCl (Y_2_Cl_2_) contains two Y atoms and, therefore, six valence electrons of Y (three per Y). Two of these electrons transfer to Cl to fill up the Cl 3*p* bands (energy < −4.5 eV). Of the remaining four electrons, two completely fill up the two spin‐split ILBs (“3”), and the other two partially occupy the four higher‐energy bands of Y 4*d* character (“1” and “2”). This is in contrast to Y_2_C where there are only as many valence electrons of Y to fill up the C 2*p* band plus the ILB. The ILBs in YCl are separated by a gap of ≈3.5 eV from the anion (Cl) bands, whereas Y_2_C has no such gap. This difference reflects the smaller interlayer distance of YCl, which increases the energy of the ILBs through the quantum confinement effect. As the ILBs in YCl are located well below *E*_F_ and fully occupied, they are not involved in transport properties. (This situation is close to the original concept of electrides as materials in which electrons serve as immobile anions.^[^ ^22^ ^]^) LaCl is isostructural and has a band structure similar to YCl except that the ILBs are at a higher energy and slightly overlap *E*_F_.^[^ ^31^ ^]^ No magnetism has been reported for LaCl.

The comprehensive screening of material databases for electrides has been conducted recently by two groups,^[^ ^33^, ^34^ ^]^ which reported a large number of Q2DEs (PrGa, CaAu, NdGa, CaSi, SrSn, BaGe, SrGe, CaGe, SrSi, LaRuSi,^[^ ^78^ ^]^ PrScGe, NdScGe, SmScSi, BaSn, HoMgSn, PrScGe, NdScGe, TbMgSn, LaScGe, TmMgSn, ErMgSn, PrScSi, SmTiGe, NdScSi, CeScSb, YTiGe, Pr_2_MgNi_2_, Pr_2_MgGe_2_, Nd_2_MgNi_2_, Sm_2_MgGe_2_, Dy_2_MgSi_2_, Ce_2_MgSi_2_, Dy_2_MgGe_2_, Mg(ScGa)_2_, Tb_2_MgNi_2_, Y_2_MgCu_2_, Li(NdSi)_2_, Yb_2_MgSi_2_, Mg_3_Sn, Pr_5_(CoB_3_)_2_, Nd_5_(CoB_3_)_2_, Ba_2_LiN,Na_3_Cl_2_, Ca_5_Ga_2_N_4_^[^ ^64^ ^]^). A more detailed account of their electronic structures is warranted.

In addition to materials already known and synthesized, efforts have been ongoing to identify unknown Q2DEs by ab initio computational techniques. Tada et al. carried out a high‐throughput search and proposed a number of candidate materials, including dialkali halides (e.g., Na_2_Cl) and Cs_2_O_1−*x*_F_*x*_.^[^ ^29^ ^]^ Ming et al. predicted, using the particle swarm optimization algorithm, that alkaline‐earth sub‐pnictogenides Sr_2_P, Ba_2_P, and Ba_2_As are Q2DEs isostructural to Ca_2_N.^[^ ^30^ ^]^ Figure 10c shows the calculated band structure of Sr_2_P. The bands highlighted in red are ILBs, as can be seen from the PED isosurface (Figure 10d) and the plane‐averaged PED (Figure 10e). Alkaline‐earth monofluorides *M*F (*M* = Ca, Sr, and Ba) with tetragonal symmetry (space group *P*4*/nmm*) exhibit *M*–2F–*M* stacking (two F ions in the same layer) and are predicted to be Q2DEs. The band structure (ILBs in red) and the PED for the ILBs are shown in Figure 10f–h.

### 3.2 Surface States

**Figure 11:**
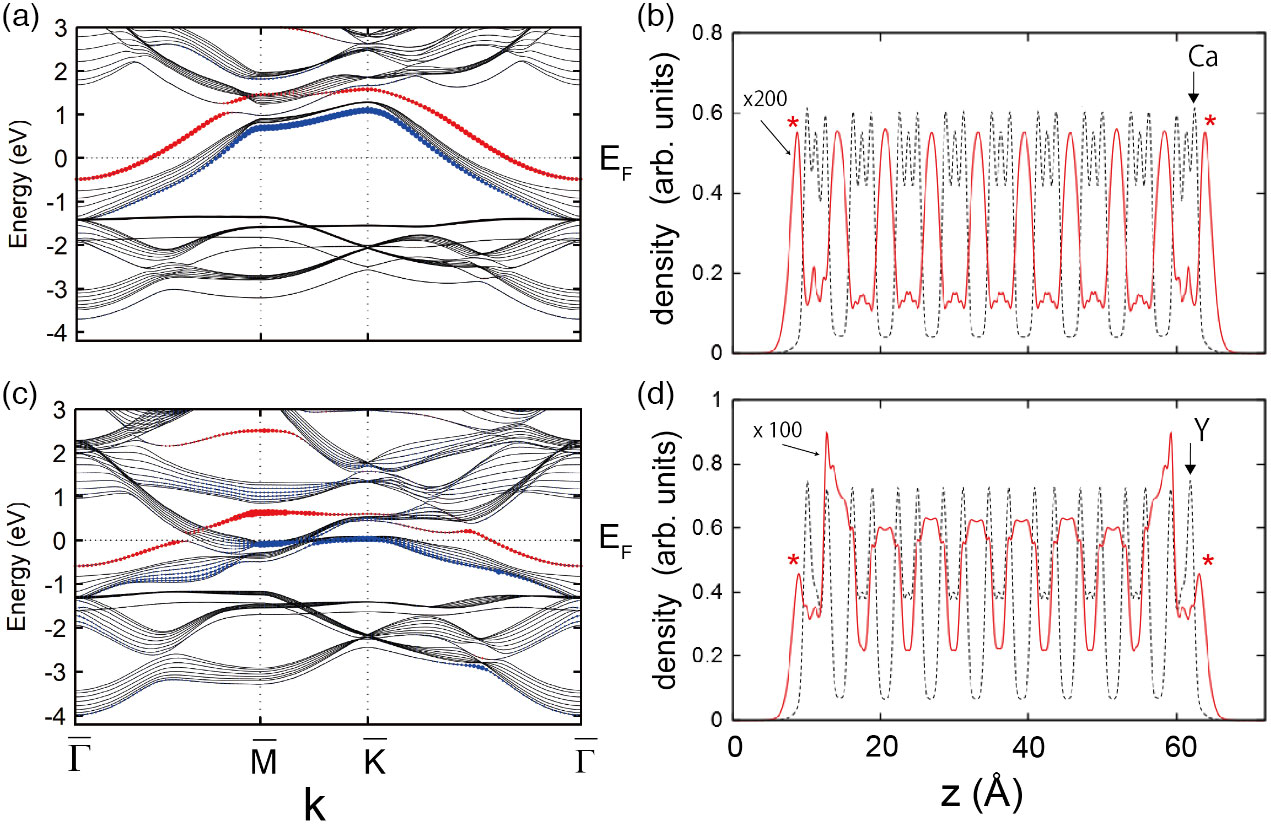
a) Energy band structure of a nine‐layer Ca_2_N slab calculated using DFT. The surface states are highlighted in red with the thickness proportional to the extra‐surface weight. b) Plane‐averaged PED (energy within 0.1 eV below *E*_F_) and total valence electron density, plotted as red solid and black dashed lines, respectively, for the same Ca_2_N slab. Each of the peaks marked by an asterisk in (b) corresponds to an extra‐surface floating state. c,d) Plots for Y_2_C corresponding to (a,b) are shown, respectively. Reproduced with permission.^[^ ^79^ ^]^ Copyright 2017, The American Physical Society.

In Section 3, we saw that floating NFE states exist on the surface of graphite and GICs. Similar floating states appear on the surface of Q2DEs.^[^ ^79^ ^]^ **Figure** **11**a shows the energy band diagram for a nine‐layer slab of Ca_2_N plotted along the symmetry directions of the surface Brillouin zone (hexagonal). (This slab is thick enough to simulate the bulk + surface states of Ca_2_N.) Here, the surface state is highlighted in red with thickness proportional to the extra‐surface weight (density outside the surface), whereas black lines show bulk energy bands.

In Figure 11b, the plane‐averaged PED for energy −0.1 eV < *E*‐*E*_F_ < 0 is plotted against *z*//*c* (red line). The total charge density (black line), whose peaks indicate the positions of the atomic layers, is also plotted here. Note that the PED near the surfaces is almost unchanged from that in the bulk (middle of the slab) except that there is an additional peak (indicated by *) outside each of the two surfaces that is separated from the atomic plane.

The appearance of such extra‐surface floating electrons in Q2DEs seems plausible from the requirement of charge neutrality. Taking Ca_2_N as an example, the crystal is essentially a stack of layers of alternating charge: ‐‐‐/[Ca_2_N]^+^/e^−^/[Ca_2_N]^+^/e^−^/‐‐‐ . If the thickness of the sample is finite and the charge distribution remains the same as in the bulk ([Ca_2_N]^+^/e^−^/‐‐‐/e^−^/[Ca_2_N]^+^), there is one more positively charged layer than negatively charged (anionic electron) layer. The extra charge should be neutralized by introducing additional electrons of charge e^−^/2 near each surface. In the case of electrides, these extra electrons tend to accumulate outside the surface rather than inside, resulting in the approximate charge distribution e^−/2^/[Ca_2_N]^+^/e^−^/‐‐‐‐‐/e^−^/[Ca_2_N]^+^/e^−/2^. We found that the extra‐surface state of Ca_2_N can be described by the LCAO method using *s*‐like localized orbitals (Wannier orbitals) located ≈1.3 Å outside of the surface. This suggests that the concept of interstitial quasi‐atoms is useful for understanding the electronic structure (both bulk and surface) of Ca_2_N and probably many other Q2DEs.

A similar partially filled floating surface state also exists on the surface of Y_2_C (Figure 11c,d), but the state is more strongly hybridized with the metal orbitals compared with Ca_2_N, resulting in a smaller density outside the surface.

### 3.3 Electrenes

**Figure 12:**
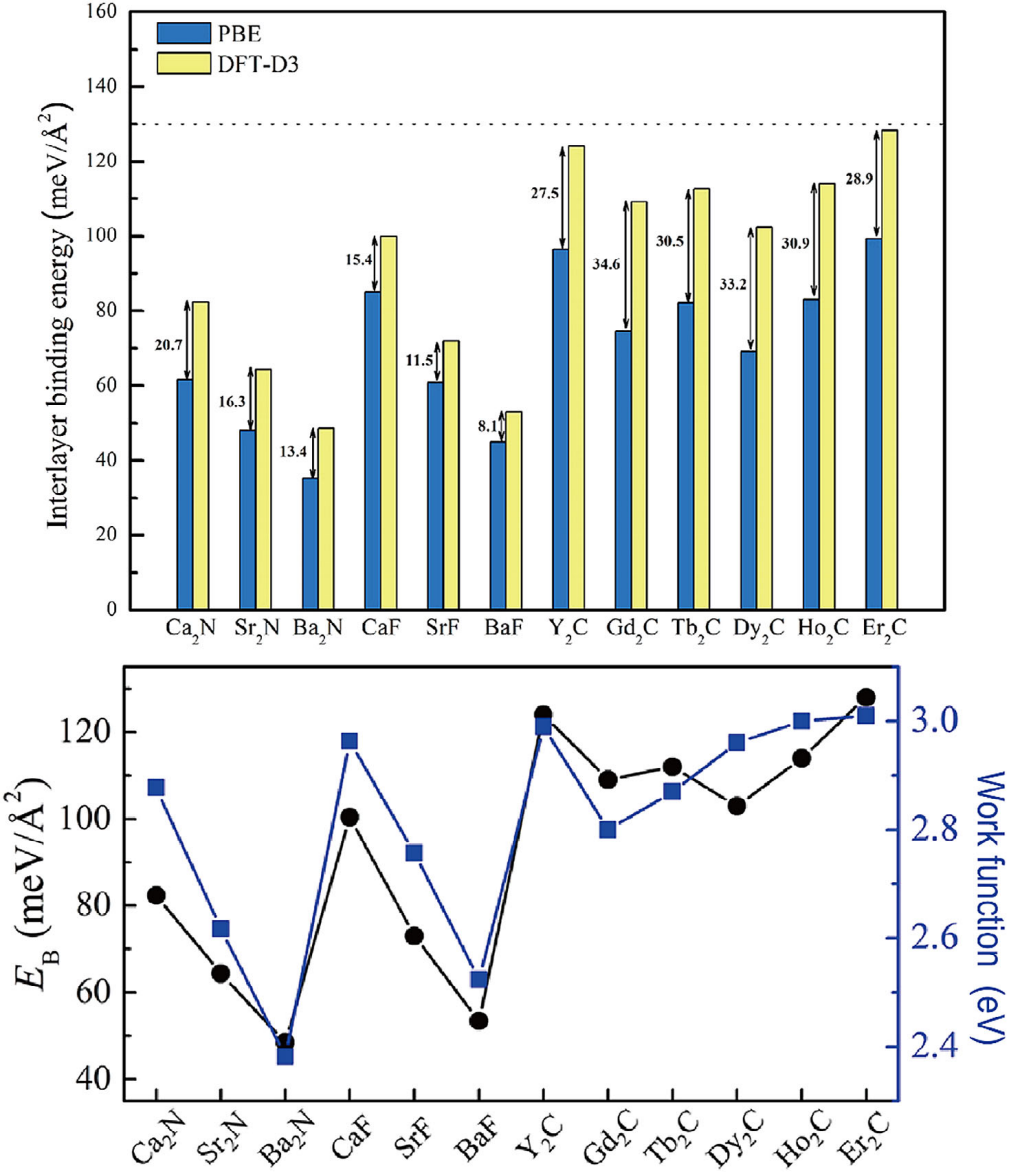
Interlayer binding energies *E*_B_ of Q2DEs calculated using DFT. In the upper panel, the yellow and blue bars show the results obtained with and without considering van der Waals corrections (DFT‐D3) to the potentials. Reproduced with permission.^[^ ^80^ ^]^ Copyright 2019, The American Chemical Society.

Much of the current interest in layered materials lies in the feasibility of thinning them down to the atomic scale. A useful parameter to gauge this feasibility is the interlayer binding energy $E_{B}=(NE_{\text{SL}}-E_{\text{bulk}})/NA$, where $E_{\text{SL}}$ and $E_{\text{bulk}}$ are the energy of a single isolated layer and the bulk, respectively, per unit cell, *A* is the base area of the unit cell, and *N* is the number of layers contained therein. **Figure** **12** shows $E_{B}$ calculated for various Q2DEs.^[^ ^80^ ^]^ The magnitude of $E_{B}$ is in the order of carbides > fluorides > nitrides. These values, although larger than those for graphite and MoS_2_ (≈20 meV Å^−2^) due to the partly ionic character of interlayer bonding, suggest that Q2DEs are exfoliable in general.

The exfoliability of Ca_2_N was demonstrated by Druffel et al., who succeeded in producing nanosheets of Ca_2_N from the bulk using liquid exfoliation: after testing 30 solvents, 1,3‐dioxolane was found to show the best performance.^[^ ^26^, ^81^ ^]^ The obtained nanosheets, which the researchers named electrenes, had the same crystal structure and stoichiometry as the bulk, and were stable for at least a month in nitrogen atmosphere or select organic solvents.

**Figure 13:**
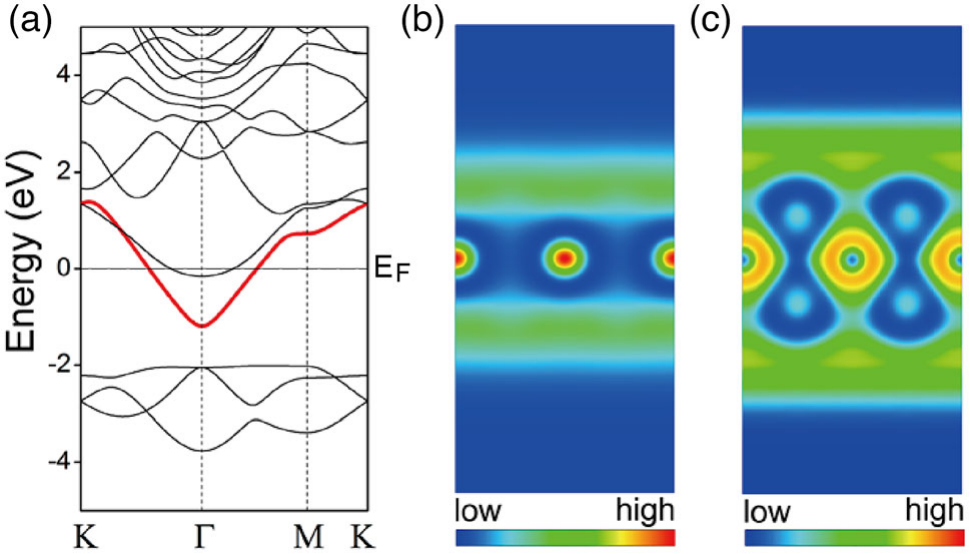
Electronic structure of Ca_2_N monolayer (electrene) calculated using DFT. a) Band structure with the lowest surface band highlighted in red. The black curve immediately above denotes the second surface band. These are the bonding and antibonding combinations of the floating electron states on the two sides of the layer, respectively. b) PED map of the lowest surface band at the Γ point. c) ELF. Reproduced with permission.^[^ ^82^ ^]^ Copyright 2014, The American Chemical Society.

The electronic structure of the Ca_2_N monolayer electrene was first calculated by Zhao et al.^[^ ^82^ ^]^ Their band structure is shown in **Figure** **13**a, where the band in red is an ILB. As shown by the PED for this band at the Γ point (Figure 13b), there are two extra‐surface floating states, one above and one below the layer plane. Obviously, these are the same surface states as those discussed in the last section for the nine‐layer slab. It is remarkable that they have survived in the monolayer limit. The two surface states are coupled via tunneling and split into bonding and antibonding states, and the red curve in Figure 13a shows the bonding state, whereas the band immediately above (in black) is the antibonding state. The system is analogous to the coupled quantum well familiar in semiconductor nanoelectronics.^[^ ^83^ ^]^ The optical transition between the two states should be sensitive to the polarization of light and the number of layers. Thus, optical absorption will be a useful tool for characterizing the quality and thickness of an electrene. The ELF is plotted in Figure 13c, which indicates that the delocalized anionic electrons and the [Ca_2_N]^+^ ionic layer form ionic bonds just like in bulk Ca_2_N.

## 4 Miscellaneous Properties of 2D Electrides and Electrenes

### 4.1 Work Functions

With anionic nonnuclear electrons confined loosely around the interstitial sites, electrides generally have very low work functions *ϕ* comparable to those of alkali metals (Na 2.8 eV; Cs 2.1 eV).^[^ ^30^, ^84^ ^]^ This property is interesting scientifically but also makes these materials useful for application as electron emitters^[^ ^85^ ^]^ and electron injection layers in thin‐film transistors (TFTs) for driving organic light‐emitting diodes (OLEDs).^[^ ^86^ ^]^

**Figure 14:**
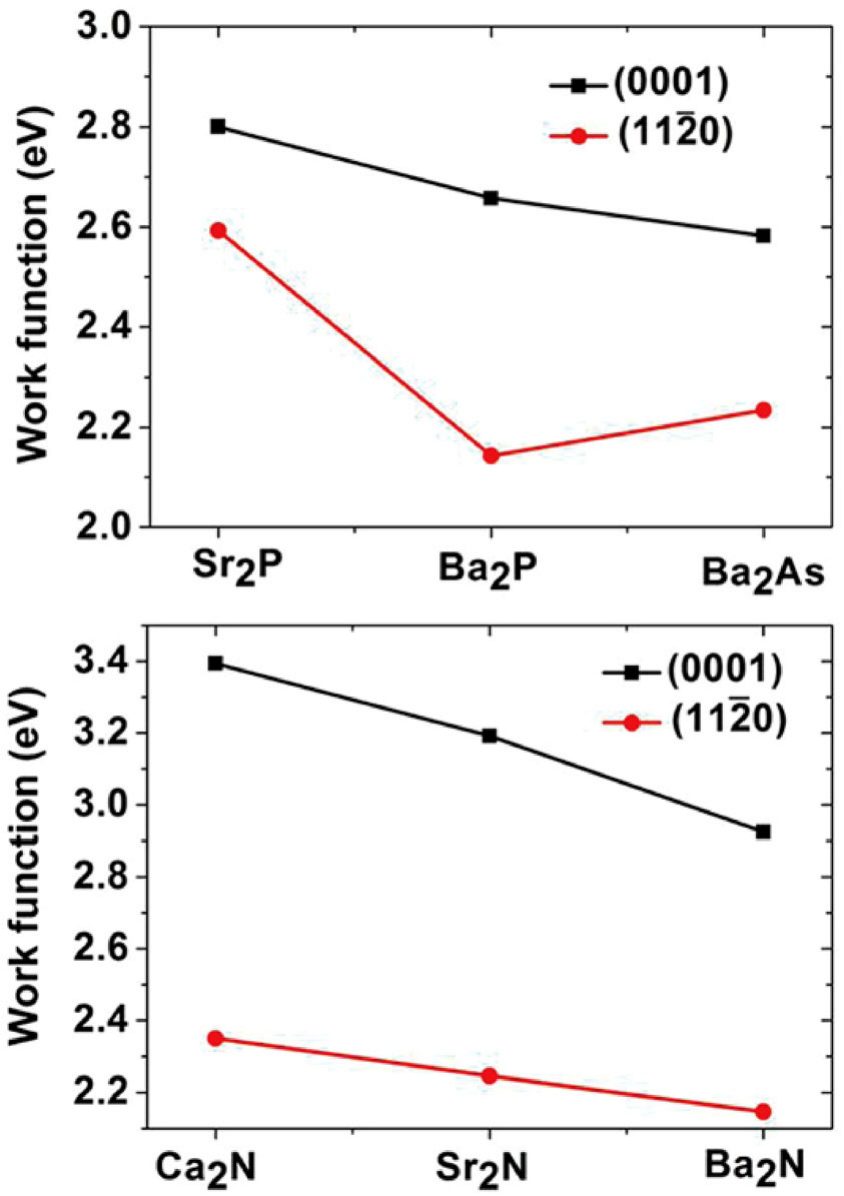
Work functions of Q2DEs calculated using DFT. Reproduced with permission.^[^ ^30^ ^]^ Copyright 2016, The American Chemical Society.

As shown in **Figure** **14**, *ϕ* is highly anisotropic and larger for the (0001) surface than for the $\left(11\bar{2}0\right)$ surface, which is perpendicular to (0001).^[^ ^30^ ^]^ Phosphides and arsenides have lower work functions than the nitrides. It is well known that the electric double layer formed as a result of the electrons seeping out of the crystal surface into vacuum enhances the work function.^[^ ^87^ ^]^ For the (0001) surface of Q2DEs, extra‐surface electrons accumulate ≈1.4 Å outside of the surface, whereas there are no such electrons outside the $\left(11\bar{2}0\right)$ surface. This difference in electric double layer formation explains the larger work function of the (0001) surface. Another mechanism that may contribute to the lower work function of the $\left(11\bar{2}0\right)$ surface is Smoluchowski smoothing,^[^ ^88^ ^]^ which lowers the work function for a corrugated surface such as $\left(11\bar{2}0\right)$.^[^ ^30^ ^]^ Note that there is a clear correlation between *ϕ* and interlayer binding energy *E*_B_, as shown in Figure 12b.^[^ ^80^ ^]^

### 4.2 Doping and Heterostructures

Doping is useful in tuning the structure and properties of matter. However, heavy doping (>10^21^ cm^−3^) without introducing significant disorder is still a challenge. With their low work function and flat (001) surface, it is expected that Q2DEs will allow the high‐density electron doping of layered materials in a clean and controlled manner.

**Figure 15:**
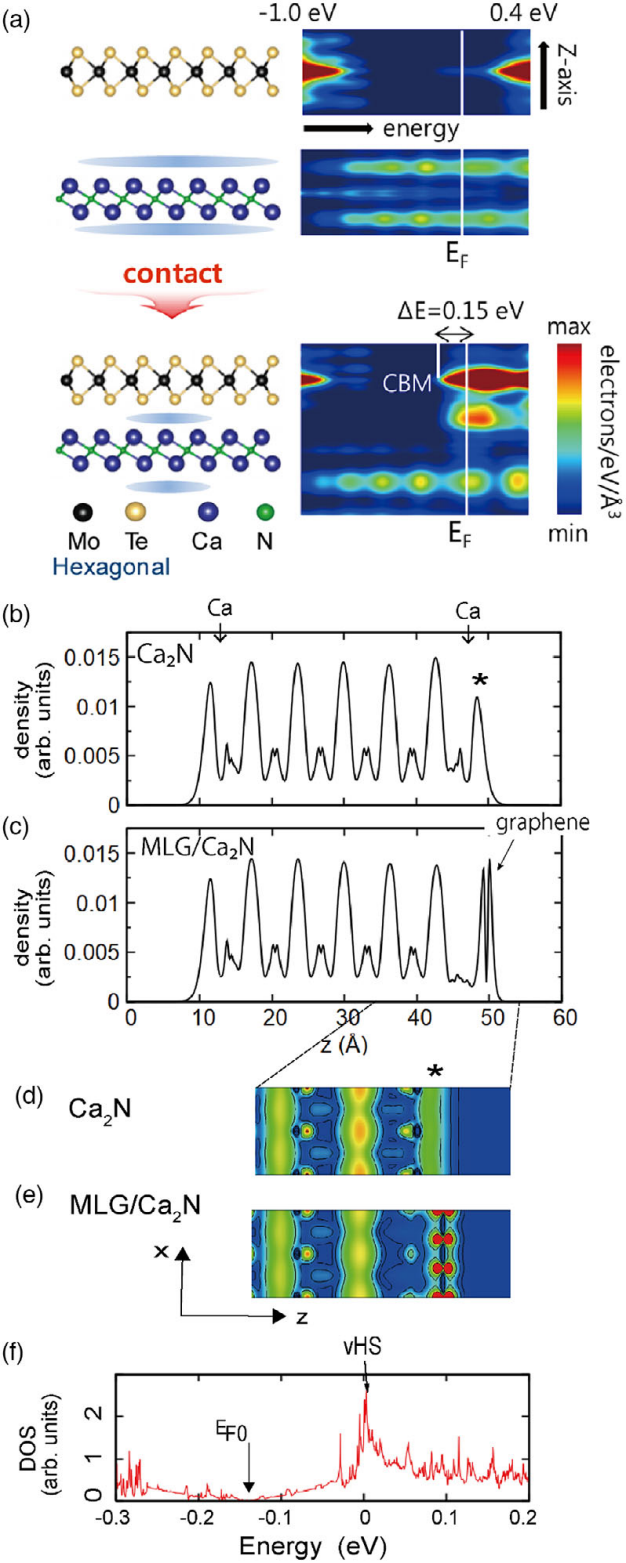
a) Local DOS for MoTe_2_/Ca_2_N calculated using DFT before (upper panel) and after (lower panel) the two materials are brought into contact. In the 2D plots on the right, the horizontal and vertical axes correspond to energy and position *z*, respectively, and the DOS is shown by color. Note that the contact lifts the Fermi level of MoTe_2_ to about 0.15 eV above the conduction band bottom. Reproduced with permission.^[^ ^89^ ^]^ Copyright 2017, The American Chemical Society. b–f) Electronic structure of a six‐layer slab of Ca_2_N with MLG placed on top. b,c) Layer‐averaged PED in the energy range −0.1 eV < *E*‐*E*_F_ < 0 for Ca_2_N and MLG/Ca_2_N, respectively. The positions of the surface Ca layers are indicated at the top of (b). Note that the extra‐surface floating electrons (indicated by *) of Ca_2_N transfer almost entirely to the π orbitals of MLG upon contact. d,e) Color maps corresponding to (b,c), respectively. f) DOS of MLG in contact with Ca_2_N. The Fermi level of free‐standing MLG (*E*_F0_) is lifted into the π* band and pinned at the vHS, where DOS diverges logarithmically. Adapted with permission.^[^ ^91^ ^]^ Copyright 2017, The American Physical Society.

Kim et al. formed a MoTe_2_/Ca_2_N heterostructure on SiO_2_ and studied electron injection into MoTe_2_.^[^ ^89^ ^]^ MoTe_2_, a semiconductor with a hexagonal (2 H) structure, undergoes a phase transition into a monoclinic (1 T′) phase and becomes a metal when electron doped above a density of n*=2.2 × 10^21^ cm^−3^ (areal density of 1.6 × 10^14^ cm^−2^).^[^ ^90^ ^]^ DFT calculation predicts that MoTe_2_ in contact with Ca_2_N is a metal with the Fermi level at 0.15 eV above the conduction band bottom and electron density of ≈10^14^ cm^−2^ (**Figure** **15**a). The researchers synthesized such heterostructures and confirmed by Raman measurements that MoTe_2_ near the interface is indeed in the monoclinic phase. They also carried out friction force microscopy for samples with various thicknesses of MoTe_2_ and confirmed that MoTe_2_ remains metallic up to a thickness of 120 nm. Combining these results, they concluded that MoTe_2_ on top of Ca_2_N is in the monoclinic phase over a distance of ≈100 nm from the interface. Does this mean that MoTe_2_ is doped to a density above n* for over ≈100 nm from the interface, whereas the conventional theory of screening says that doping extends only up to the Thomas–Fermi screening length of ≈1 nm? Further study is warranted to answer this question.

Inoshita et al. studied the electron doping of graphene in contact with Ca_2_N by DFT calculation.^[^ ^91^ ^]^ The result for MLG shows that most of the electrons in the extra‐surface state of Ca_2_N (indicated by asterisks in Figure 15b,d) are transferred to the π* band of graphene, whereas the electron density inside Ca_2_N remains largely intact (Figure 15c,e). The charge transfer dopes the graphene to an extremely high density of 5 × 10^14^ cm^−2^, causing *E*_F_ to be pinned to the 2D van Hove singularity (vHS) in the π* band, where the DOS diverges logarithmically (Figure 15f). What makes this singularity special is that it is a saddle point where the dispersion is both electron‐like and hole‐like. A number of exotic phenomena have been predicted to occur when *E*_F_ is at or near this type of singularity, including superconductivity (*p *+ i*p*‐wave,^[^ ^92^, ^93^ ^]^ *d*‐wave,^[^ ^94^, ^95^, ^96^ ^]^ *f*‐wave^[^ ^97^ ^]^), ferromagnetism,^[^ ^94^, ^98^ ^]^ antiferromagnetism,^[^ ^94^ ^]^ spin density wave,^[^ ^99^ ^]^ and charge density wave.^[^ ^100^ ^]^ The study of these effects has been hampered, because traditional methods of heavy doping such as chemical doping introduce significant disorder.

Interfacing a Q2DE with a layered material generally results in charge transfer and enhances their mutual bonding through Coulomb attraction. As a result, the bond length between the materials across the interface becomes typically ≈2 Å, which is ≈1 Å smaller than that for van der Waals interactions (3–4 Å) but ≈1 Å larger than that for covalent or ionic bonds. Woomer et al. stressed the importance of such intermediate‐length bonds, unseen in conventional materials, and named these new heterostructures *donor*–*acceptor heterostructures*.^[^ ^101^ ^]^ The length of such bonds (quasi‐bonds) depends on the amount of charge transfer, steric hindrance (e.g., lattice mismatch), and structural relaxation at the interface. The control of such parameters will enable us to tailor the properties of donor–acceptor heterostructures, including sliding friction and work function.

### 4.3 Optical Properties

The strong anisotropy of the ILBs leads to attractive optical properties. The dielectric function tensor $\overset{═}{\varepsilon }(\omega )$ for Ca_2_N (point group *D*_3*d*_) has two independent components, $\varepsilon_{x}$ and $\varepsilon_{z}$, where *z* (*x*) is parallel (perpendicular) to the crystalline *c*‐axis. From this, one can derive the dispersion relation for the transverse magnetic (TM) wave propagating in the *xz* plane of the crystal as 
(1)

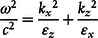

In ordinary materials, $\varepsilon_{x}$ and $\varepsilon_{z}$ have the same sign, but there are also materials, called hyperbolic materials, for which they have different signs in some frequency range.

**Figure 16:**
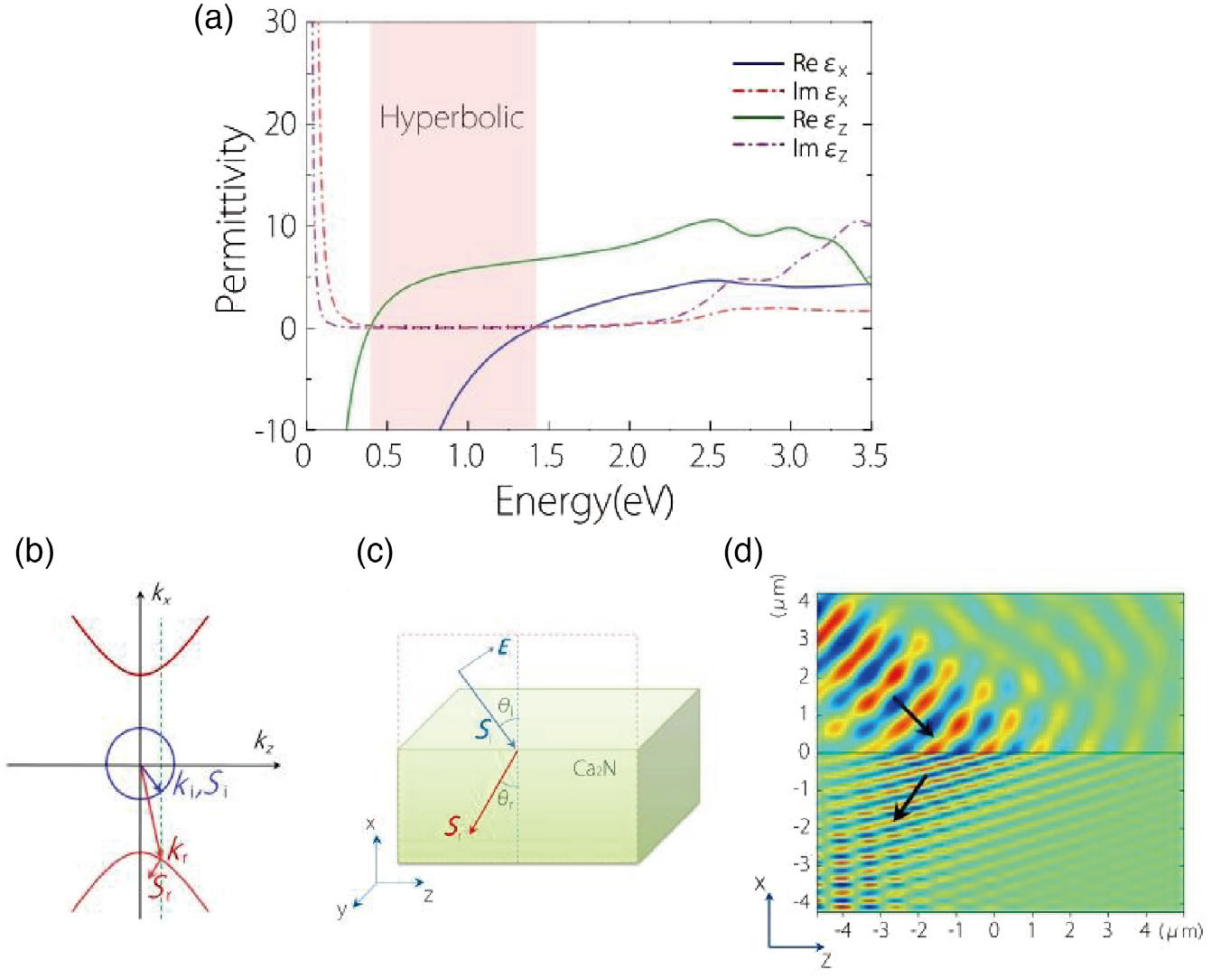
a) Principal components of the dielectric tensor (permittivity) of Ca_2_N. The material is hyperbolic in the frequency region shown by the shaded area. b) EFCs given by Equation (1). The blue circle and red hyperbola show the EFC for vacuum and Ca_2_N, respectively. Here, ***k***_i_ (***k***_r_) is the wave vector of the incident (refracted) wave, whereas ***S***_i_ and ***S***_r_ are the corresponding Poynting vectors. c) Schematic of negative refraction at a vacuum/Ca_2_N interface. The crystal *c*‐axis is parallel to the *z*‐direction. d) Simulation result of light propagation across vacuum/Ca_2_N interface. The color indicates the electric field strength, and the arrows show Poynting vectors. Reproduced with permission.^[^ ^102^ ^]^ Copyright 2017, The American Physical Society.

Guan et al.^[^ ^102^, ^103^ ^]^ theoretically examined the dielectric function of Ca_2_N, calculating the interband contribution from the DFT band structure (Figure 6b) while treating the intraband contribution in the Drude approximation, which neglects electron–electron interactions. The study revealed that Ca_2_N is hyperbolic over a wide frequency range spanning short‐wavelength IR to near IR (0.38–1.40 eV in energy; 3.3 μm to 880 nm in wavelength), shown by the shaded area in **Figure** **16**a. Interestingly, the imaginary part of $\overset{═}{\varepsilon }(\omega )$ is very small over the entire hyperbolic window, indicating the strong suppression of damping. This low damping results from the fact that there is no energy band except the ILB in the wide energy window of *E*‐*E*_F_ = −1.5 to 1 eV (Figure 6b), precluding interband transitions. Sr_2_N and Ba_2_N possess hyperbolic windows in the ranges of 0.4–1.25 eV and 0.31–0.96 eV, respectively. Few‐layer electrenes of Ca_2_N and Sr_2_N are also hyperbolic, the hyperbolicity being tunable by changing the number of layers or applying a uniaxial pressure.

Many unusual properties of hyperbolic materials ensue from Equation (1), which implies that the equifrequency contour (EFC) in the *k* (wave vector) space is a circle in air ($\varepsilon_{x}=\varepsilon_{y}$), as shown by the blue curve in Figure 16b, whereas it is a hyperbola in a hyperbolic material ($\varepsilon_{x}/\varepsilon_{y}<0$) (red curves in Figure 16b). Taking the interface to be parallel to *z* and perpendicular to *x* (Figure 16c), the *z* component of ***k*** is conserved during refraction. For a given incident wave vector ***k***_i_, the wave vector of the refracted wave ***k***_f_ is determined as shown in the figure. Because the Poynting vector ***S*** is normal to the EFC, ***S*** for the incident wave ***S***_i_ and that for the refracted wave ***S***_r_ must be on opposite sides of the normal to the interface, implying a negative refraction for an arbitrary incident angle. Guan confirmed this negative refraction by a numerical simulation of light propagation for air/Ca_2_N (Figure 16d).

The hyperbolic materials known to date are mostly artificial metamaterials.^[^ ^104^ ^]^ If we can achieve hyperbolicity over a wide wavelength range and with small loss using naturally existing materials instead of artificial metamaterials, it will enable a wide range of applications.^[^ ^105^ ^]^ While Q2DEs are promising as hyperbolic materials, hyperbolicity with low loss is not unique to these materials, as there are also other layered materials such as MoS_2_ showing favorable hyperbolicity.^[^ ^106^, ^107^ ^]^

When a metal and an insulator are in contact, oscillations of electronic charge density confined to the interface, called surface plasmons, can be excited if the real parts of the dielectric functions of the two materials have different signs.^[^ ^108^ ^]^ Calculations for Ca_2_N/SiO_2_ and Sr_2_N/SiO_2_ structures show that both systems sustain surface plasmons in near IR (1.1–1.2 eV), which is lower in energy than for conventional systems (≈3.4 eV for Al/SiO_2_). Although the energy of surface plasmons in electrenes does not differ significantly from that of bulk electrides, the wavelength and confinement size are smaller by two orders of magnitude, making electrenes promising materials for nanoplasmonics.

The calculation of the plasmon dispersion requires the inclusion of electron–electron interactions neglected in the Drude approximation. A theory developed by Cudazzo and Gatti based on the random‐phase approximation (RPA) revealed that the bulk plasmon in Ca_2_N has a negative dispersion (the frequency decreases as the wavelength *q* increases from 0).^[^ ^109^ ^]^ This result, which is in contrast with the positive dispersion $\omega \propto \sqrt{q}$ of plasmons in a 2D free electron gas, implies that the ILB in Ca_2_N is not as free electron‐like as we would expect from the band structure.

### 4.4 Electron–Phonon Interactions and Transport Properties

The electron–phonon interactions in a Ca_2_N monolayer were analyzed using DFT by Zeng et al., who found that the electron–phonon matrix elements are small for all phonon modes compared with other 2D materials such as graphene.^[^ ^110^ ^]^ The dominant interaction originates from long‐wavelength longitudinal acoustic (LA) modes, but the optical modes near the Brillouin zone boundary interact strongly as well. The researchers calculated the phonon‐limited mobility using the Boltzmann equation and obtained the value of 189 cm^2^ V^−1^s^−1^ at 300 K. This value, which is significantly larger than the mobility of good metals (≈30 cm^2^ V^−1^s^−1^ for Cu^[^ ^111^ ^]^), is in good agreement with the experimental value of 160 cm^2^ V^−1^s^−1^.^[^ ^27^ ^]^ The mobility markedly increases, as the conduction electron density decreases (hole doping) from the intrinsic density of 8.9 × 10^14^ to 1.3 × 10^14^ cm^−2^, where the mobility attains a maximum value of about 3000 cm^2^  V^−1^s^−1^. (This corresponds to a downshift of *E*_F_ by 1 eV.) This increase in mobility by hole doping is explained as due to the reduced *k* space for scattering satisfying energy and momentum conservation.

The nondimensional electron–phonon coupling parameter *λ* of the Ca_2_N monolayer is estimated to be 0.78, which is considerably larger than that for *sp* metals (0.18 for Na,^[^ ^112^ ^]^ 0.44 for Al,^[^ ^113^ ^]^ and 0.14 for Cu^[^ ^114^ ^]^) or graphene (0.21^[^ ^115^ ^]^). Even though *λ* considers all phonon modes equally, unlike mobility, which emphasizes the contribution from lower frequency phonons with energy <kT, this large *λ* value is rather surprising. From this *λ*, the superconducting transition temperature *T*_c_ is estimated using McMillan's formula^[^ ^116^ ^]^ to be 4.7 K, assuming Coulomb pseudopotential μ* = 0.1. A similar calculation for the Y_2_C monolayer by Ge et al. gave *λ* = 0.53 and *T*_c_ = 0.9 K.^[^ ^117^ ^]^ The calculated phonon linewidths and Eliashberg spectral function *α* ^2^ *F*(*ω*) indicate that the main contribution comes from high‐frequency optical phonons with energy >300 cm^−1^. Thus far, there has been no report on the observation of superconductivity in Q2DEs.

As anionic electrons are nucleus‐free, one would intuitively expect their interaction with less polar phonons (mainly acoustic phonons) to be weak owing to the short‐range nature of such interactions, whereas long‐range interactions with polar phonons such as longitudinal‐optical (LO) modes are considered to be still strong. However, the above‐mentioned results seem to suggest that this expectation is too naïve.

### 4.5 Topological Electrides

**Figure 17:**
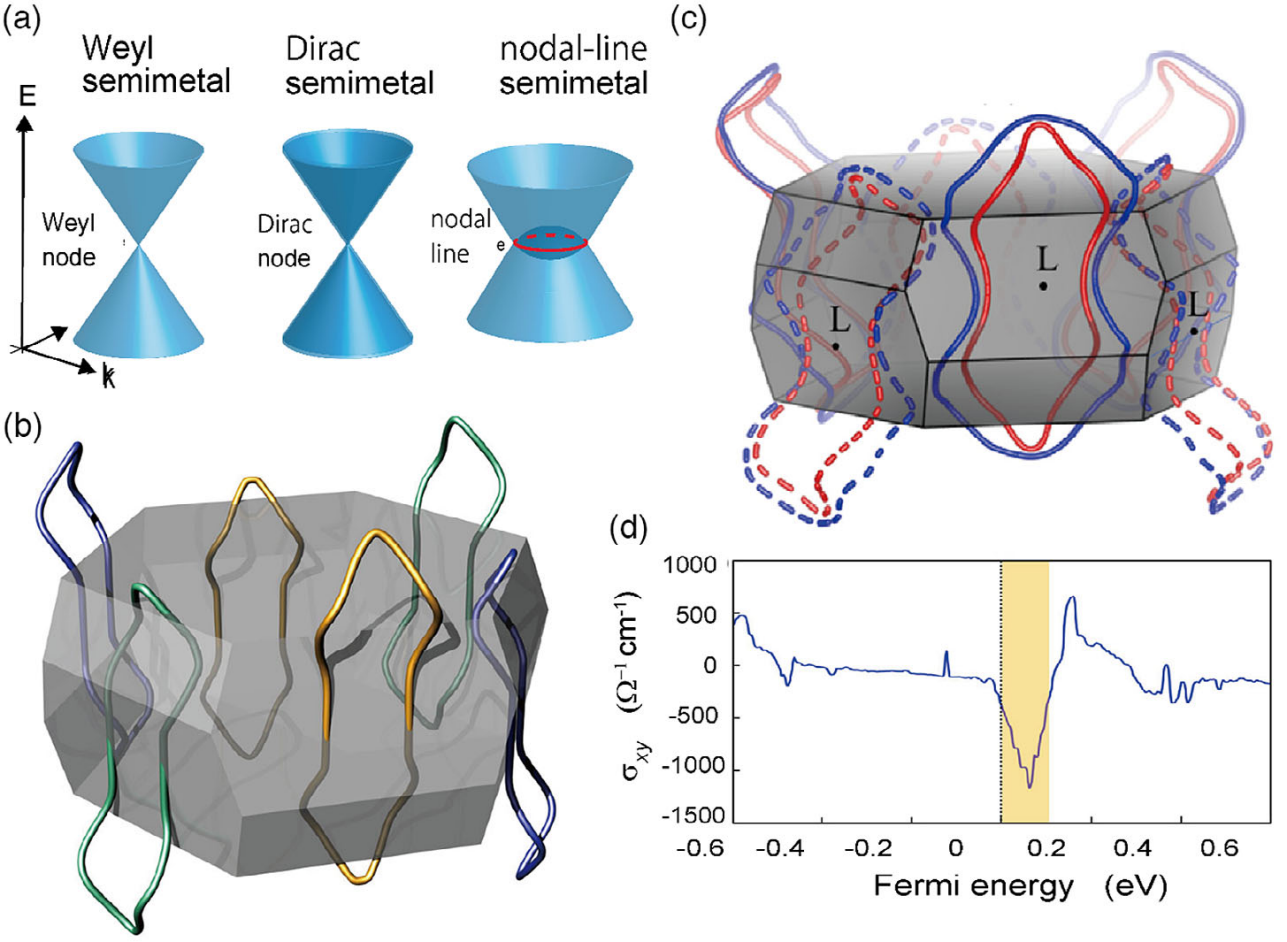
a) Classification of topological semimetals. Reproduced with permission.^[^ ^119^ ^]^ Copyright 2018, The Physical Society of Japan. b) Dirac nodal lines in the first Brillouin zone calculated for Y_2_C using DFT. Reproduced under the terms of the CC‐BY 4.0 license.^[^ ^120^ ^]^ Copyright 2018, The Authors, Published by The American Physical Society. c) Weyl nodal lines in the first Brillouin zone calculated for ferromagnetic Gd_2_C using DFT. d) Fermi energy dependence of the anomalous Hall conductivity of Gd_2_C. c,d) Reproduced with permission.^[^ ^123^ ^]^ Copyright 2020, The American Physical Society.

Recent years have witnessed a surge of interest in materials in which two energy bands cross, without gapping, near *E*_F_ at a set of isolated or continuous points in *k* space. These gapless materials (semimetals) can be classified into 1) Dirac semimetals in which each of the crossing bands is doubly degenerate including spin, resulting in fourfold degeneracy at the crossing point, and 2) Weyl semimetals in which the bands are nondegenerate, resulting in twofold degeneracy at the crossing point (**Figure** **17**a).^[^ ^118^, ^119^ ^]^

As the bands usually disperse linearly near a crossing point, Dirac/Weyl semimetals exhibit high mobility and broadband optical absorption, which may find application in electronic and optical devices. However, what sparked the broad interest in these materials is the topological nature of such band crossings; i.e., the crossings result in the nontrivial connectivity of the wave functions (both bulk and surface) in *k* space, leading to exotic phenomena, such as the giant magnetoresistance, anomalous Hall effect, anomalous thermoelectric effect, and magneto‐optical Kerr effect. With topological semimetals having favorable electronic structures still limited in number, there is a strong demand for useful design principles.

Against this background, Hirayama et al. proposed that electrides offer a fertile platform for topological material research.^[^ ^120^ ^]^ As the low work function of an electride causes an anionic electron (interlayer) band to appear near *E*_F_, the band is likely to overlap with ordinary atomic‐orbital‐derived bands to produce topological band crossings. They demonstrated this proposal by DFT calculations, identifying four topological electrides including Y_2_C (the other three are Sc_2_C, Sr_2_Bi, and HfBr).

As discussed in Section 3.1, Y_2_C is a two‐band system with an Y 4*d*‐like band and an ILB crossing near *E*_F_ on the L‐B_1_ line (indicated by a circle in Figure 7a) in the Brillouin zone. Interestingly, this degeneracy is found to occur not only at isolated points but along closed continuous loops in *k* space (Dirac nodal lines; right panel of Figure 17a), as shown in Figure 17b. In general, topological semimetals have surface states associated with their bulk band crossings. Thus, the surface state of Y_2_C (Figure 11c) is a topological surface state that originates from the Dirac nodal lines in the bulk. The topological electronic structure of Y_2_C was also studied by Huang et al.^[^ ^121^ ^]^ (paramagnetic phase) and Liu et al. (ferromagnetic phase).^[^ ^122^ ^]^

When a material is magnetized, a spin‐degenerate band is split into two, and therefore, a Dirac node is split into two Weyl nodes. Liu et al. examined the topology of the electronic structure of the ferromagnetic electride Gd_2_C and showed that it hosts Weyl nodal lines shown in Figure 17c.^[^ ^123^ ^]^ (Similar Weyl nodal lines were obtained for ferromagnetic Y_2_C in the previous study^[^ ^122^ ^]^) One of the signatures of Weyl semimetals is the anomalous Hall effect. A large anomalous Hall conductivity exceeding 1000 Ω^−1^ cm^−1^ is predicted for Gd_2_C if its Fermi level is lifted by 60 meV, as shown in Figure 17d.

In most topological materials reported to date, the underlying band overlap originates from spin–orbit interactions and, therefore, can take place only in materials containing heavy elements. Topological electrides offer a significant advantage in that they do not require spin–orbit interactions to have band crossings and, therefore, heavy elements, which are often costly or hazardous.

### 4.6 Surface Modification

Electrides are generally chemically unstable in ambient atmosphere. This drawback should be overcome to utilize their functionalities and properties. Faseela et al. attempted to passivate Ca_2_N by forming a Ag layer on its surface using a wet chemical process.^[^ ^124^ ^]^ The produced material was an assembly of Ca_2_N fine particles covered with 55 nm‐thick silver, which was found to be stable for up to 17 min in ambient air. The work function after passivation was 2.78 eV, comparable to that of Ca_2_N (2.4 eV for $\left(11\bar{2}0\right)$ and 3.4 eV for (0001)^[^ ^30^ ^]^) and significantly smaller than that of metallic Ag (4.7 eV).

**Figure 18:**
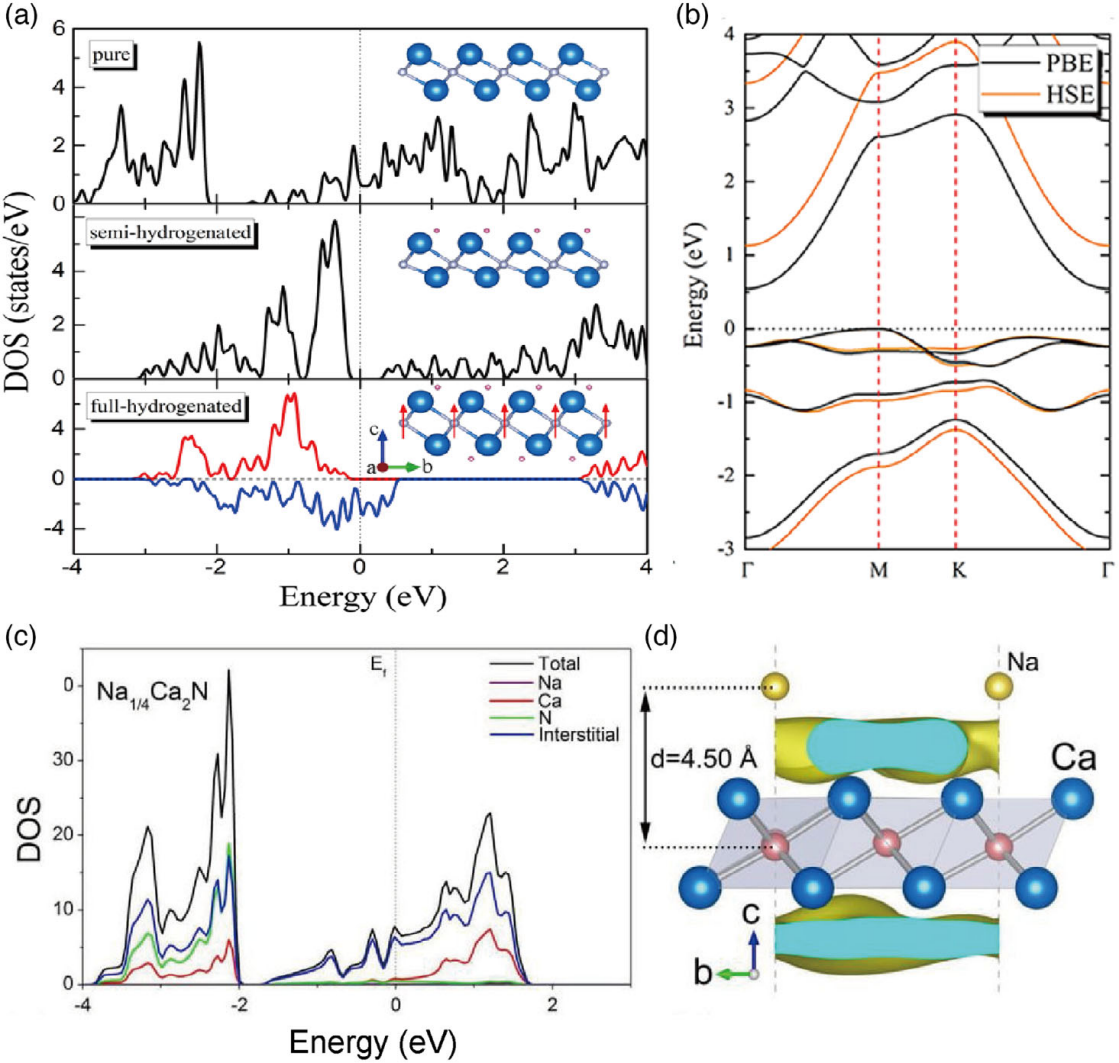
a) DOS calculated for (upper panel) Ca_2_N monolayer, (middle panel) semihydrogenated Ca_2_N monolayer, and (lower panel) fully hydrogenated Ca_2_N monolayer. b) Band structure of semihydrogenated Ca_2_N monolayer calculated using DFT with the PBE functional (black) and HSE functional (red). a,b) Reproduced with permission.^[^ ^125^ ^]^ Copyright 2019, The American Chemical Society. c) Total and PDOS calculated for Ca_2_N monolayer after Na absorption. d) PED isosurface for Na‐absorbed Ca_2_N monolayer calculated for an energy window of −0.05 eV < *E*‐*E*_F_ < 0.05 eV. c,d) Reproduced with permission.^[^ ^126^ ^]^ Copyright 2015, The American Chemical Society.

Qiu et al. studied, by DFT calculation, the functionalization of a Ca_2_N monolayer by hydrogen deposition.^[^ ^125^ ^]^ When one side of a monolayer is fully covered with H (semihydrogenation), all the anionic electrons transfer to hydrogen, and the Fermi level drops to the top of the valence band of N 2*p* character (middle panel of **Figure** **18**a (DOS) and Figure 18b [band structure]). The system is a nonmagnetic semiconductor with a band gap of 0.54 eV (calculated with the Perdew–Burke–Ernzerhof (PBE) functional) or 1.13 eV (calculated with the Heyd–Scuseria–Ernzerhof (HSE) hybrid functional). The hydrogenation of both sides of the monolayer leads to a nesting‐induced structural change, extending the unit cell to 3×3. The Fermi level enters the flat valence band of Figure 17b, and the system becomes a ferromagnetic half‐metal with a magnetic moment of 1 μ_B_ per unit cell (bottom panel of Figure 18a). The flatness of the valence band (i.e., large DOS) is believed to play a key role in bringing about this ferromagnetism. The Curie temperature is estimated to be 194 K.

The absorption of alkali metals on electrenes is interesting not only for application but also in terms of fundamental science. According to DFT calculations, among Li, Na, K, Be, Mg, Ca, and Al, the only elements that are stable on the surface of Ca_2_N (and also Sr_2_N) are K and Na.^[^ ^126^ ^]^ The DOS of Ca_2_N after Na absorption (Figure 18c) shows that the system is metallic with the electron states at the Fermi level contributed mainly by anionic electrons (indicated as interstitial in the figure). More interestingly, the Na ions are absorbed outside of the anionic electron sheets (Figure 18d). The energy barrier for the diffusion of Na was found to be very small (0.08 eV), which may be due to the smoothness of the electron sheet over which Na diffuses. Similar results were obtained for Na on Y_2_C.^[^ ^127^ ^]^

### 4.7 Interlayer Sliding

Having high‐density 2D electrons in the interlayer space strongly affects the interlayer sliding of Q2DEs. Yi et al. compared the band structures of bulk and bilayer Ca_2_N for various layer stacking and found the results to be insensitive to stacking.^[^ ^128^ ^]^ Furthermore, as layers are slid against each other in lateral directions, the band structure remains almost invariant. The complete screening of the cationic layers by anionic electrons is suggested to explain the insensitivity of the electronic structure to stacking/sliding. The interlayer sliding of Ca_2_N has also been studied by Wang et al.,^[^ ^129^ ^]^ who pointed out that Ca_2_N has a larger interlayer binding energy but a lower interlayer friction than van der Waals‐bonded layered materials, such as graphite, h‐BN, and MoS_2_.

### 4.8 Pressure Effects

**Figure 19:**
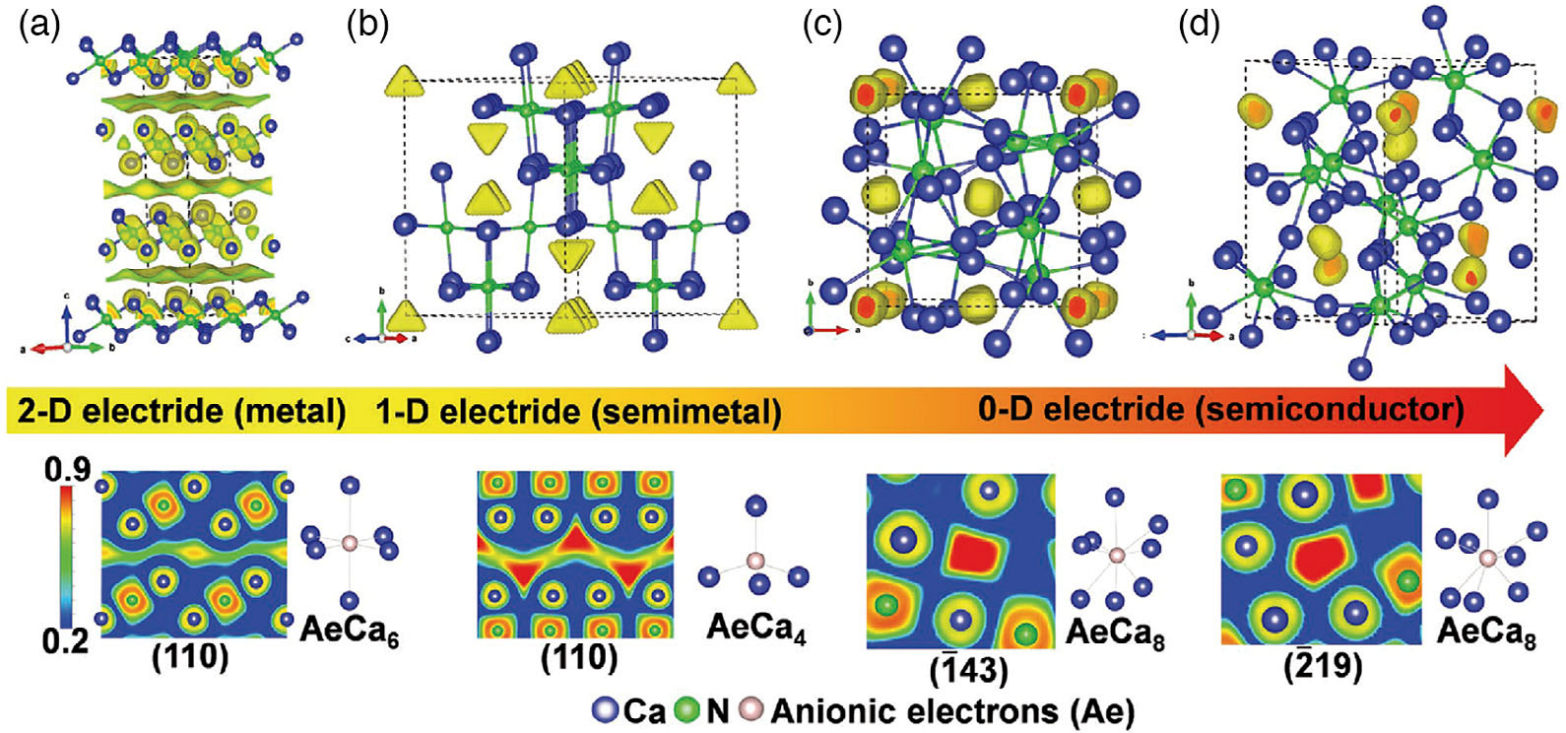
Evolution of the crystal structure and ELF of Ca_2_N calculated using DFT under increasingly strong hydrostatic pressure. The space groups are a) $R\bar{3}m$, b) $Fd\bar{3}m$, c) $I\bar{4}2d$, and d) *Cc*. Reproduced under the terms of the CC‐BY 4.0 license.^[^ ^132^ ^]^ Copyright 2018, The Authors, published by Wiley‐VCH.

Applying hydrostatic pressure to a Q2DE initially compresses the crystal in the *c*‐direction,^[^ ^130^ ^]^ but a further increase in pressure will trigger nontrivial phase transitions, as shown in **Figure** **19**.^[^ ^130^, ^131^, ^132^ ^]^ A combined computational–experimental study for *AE*_2_N (*AE* = Ca, Sr, and Ba) showed that the low‐pressure rhombohedral phase ($R\bar{3}m$) transforms into a cubic phase ($Fd\bar{3}m$) at 2.8 GPa.^[^ ^132^ ^]^ The transformation causes the ILB to disappear, forcing the excess electrons out of the interlayer space to channel‐like voids. The resulting material is a quasi‐1D electride with a semimetallic band structure. With a further increase in pressure, the system changes to a tetragonal $I\bar{4}2d$ phase at 13.5–20.5 GPa and then to a monoclinic *Cc* phase, both of which are quasi‐0D electrides. These are semiconductors with *E*_F_ located at the top of a flat valence band, separated from the conduction band by an energy gap. The electrical resistance shoots up by seven orders of magnitude as a result of these phase transformations. The lower panels of Figure 19 show how the ELF evolves with pressure. Note that Ca_2_N changes from a metal to an insulator with increasing pressure, contrary to the reverse trend observed in ordinary materials.

## 5 Outlook and Conclusion

ILBs that exist in layered materials above *E*_F_ drop to cross *E*_F_ in cation‐intercalated compounds or in electrides consisting of ionic layers with a positive net charge, resulting in a host of interesting electronic properties. This downshift of *E*_F_ is governed by the electrostatic potential within the interlayer space and the interlayer distance. If such a material is in contact with another material, structural relaxation at the interface may also affect the energy of ILBs.^[^ ^101^ ^]^

As the anionic electrons in the interlayer space are nucleus free, they are expected to interact only weakly with phonons (at least nonpolar phonons). The high mobility of Ca_2_N^[^ ^27^ ^]^ seems to support this expectation. However, we still do not know how general this statement is. The effect of long‐range polar phonons on the interlayer electrons remains unexplored. In connection with this, it is intriguing that the Ca_2_N electrene has a rather large *λ* of 0.78, although the electron–phonon matrix elements, including those for the LO mode, are small.^[^ ^110^ ^]^ In view of this large *λ* value of Ca_2_N and the high *T*_c_ of 39.3 K predicted for the Li_6_P electride under pressure,^[^ ^133^ ^]^ superconductivity will continue to be an interesting area of ILB research. As the multiple‐band structures play an essential role in the superconductivity of Li_6_P and C_6_Ca, promising materials will be those with multiple conduction bands. The softening of polar phonons or the use of ions with a larger charge state or polarizability may also help.

Interlayer electrons are localized around interstitial sites and, at the same time, spread out as itinerant electrons. This localization/delocalization duality manifests itself in magnetism, where localization tends to produce a localized magnetic moment while delocalization leads to exchange interactions between the moments to produce long‐range order. The degree of localization varies considerably over materials and can be tailored through materials design. This was exemplified by a recent experiment by Park et al., who found that the partial replacement of Y by Sc, having a smaller ionic radius, in Y_2_C increases the degree of anionic electron localization, resulting in larger magnetic moments.^[^ ^134^ ^]^ On a different note, the nucleus‐free nature of interlayer *s*‐like electrons implies a long spin relaxation time, which may be useful for application in spintronics.

ILB research has, thus far, focused on materials in which the ILB intersects the *E*_F_ and dominates properties such as electronic conduction. Materials with an ILB above *E*_F_ have largely escaped attention, although such an empty ILB may affect optical properties, electron emission, and electron energy relaxation. In an attempt to fill this gap, Yang et al. have recently made a high‐throughput search for 2D semiconducting materials having an ILB with an energy of 0.3–1.5 eV above *E*_F_ and identified 12 candidates.^[^ ^135^ ^]^ They proposed the use of stress to lower the energy of the ILB and induce a metal–semiconductor transition in these materials.

In the Q2DEs discussed in the present review, the ILB is positioned between narrow anion‐derived bands located on the low‐energy side and a spaghetti of cationic bands on the high energy side (Figure 6b and 7a). This characteristic energy structure implies that the properties of the material may be markedly changed by the control of *E*_F_. The hydrogenation of the Ca_2_N electrene, which changes the metal into a semiconductor upon semihydrogenation and into a ferromagnetic metal upon full hydrogenation, is an example of such control. It strikes one as odd that the topmost valence band is extremely flat in many of the Q2DEs including Ca_2_N and Y_2_C. This flatness is the key to the emergence of ferromagnetism in fully hydrogenated Ca_2_N. The origin of this flatness and its possible correlation with the ILB deserves looking into. (Flat dispersions are also observed in the phonon spectrum of Ca_2_N and Y_2_C electrenes. See Figure S3, Supporting Information, of the previous study.^[^ ^80^ ^]^)

The ability of the interlayer space of layered materials to take in and release atoms or molecules has been studied intensively for decades. In recent years, such a space has been utilized in secondary batteries. Layered materials can be easily exfoliated to produce soft materials, even if they are hard in bulk form, which makes them promising for application in flexible electronics. The involvement of interlayer floating electrons may lead to significant improvements of the properties of these layered materials.

Any material—perfect or imperfect and layered or nonlayered—has voids inside. By tuning the size and shape of the voids, or the types of atom constituting the walls surrounding the voids and their charge states, one can alter the energy of the floating electrons in the voids. This new approach, which may be termed *void electron engineering*, is likely to gain increasing importance in materials design.

## Conflict of Interest

The authors declare no conflict of interest.
